# Subcutaneously administered tirzepatide vs semaglutide for adults with type 2 diabetes: a systematic review and network meta-analysis of randomised controlled trials

**DOI:** 10.1007/s00125-024-06144-1

**Published:** 2024-04-13

**Authors:** Thomas Karagiannis, Konstantinos Malandris, Ioannis Avgerinos, Athina Stamati, Panagiota Kakotrichi, Aris Liakos, Despoina Vasilakou, Nikolaos Kakaletsis, Apostolos Tsapas, Eleni Bekiari

**Affiliations:** 1 Clinical Research and Evidence-Based Medicine Unit, Second Medical Department, Aristotle University of Thessaloniki, Thessaloniki, Greece; 2 Diabetes Centre, Second Medical Department, Aristotle University of Thessaloniki, Thessaloniki, Greece; 3 School of Medicine, Faculty of Health Science, Aristotle University of Thessaloniki, Thessaloniki, Greece; 4 Harris Manchester College, University of Oxford, Oxford, UK

**Keywords:** GIP/GLP-1 receptor agonist, GLP-1 receptor agonist, Network meta-analysis, Semaglutide, Systematic review, Tirzepatide

## Abstract

**Aims/hypothesis:**

We conducted a systematic review and network meta-analysis to compare the efficacy and safety of s.c. administered tirzepatide vs s.c. administered semaglutide for adults of both sexes with type 2 diabetes mellitus.

**Methods:**

We searched PubMed and Cochrane up to 11 November 2023 for RCTs with an intervention duration of at least 12 weeks assessing s.c. tirzepatide at maintenance doses of 5 mg, 10 mg or 15 mg once weekly, or s.c. semaglutide at maintenance doses of 0.5 mg, 1.0 mg or 2.0 mg once weekly, in adults with type 2 diabetes, regardless of background glucose-lowering treatment. Eligible trials compared any of the specified doses of tirzepatide and semaglutide against each other, placebo or other glucose-lowering drugs. Primary outcomes were changes in HbA_1c_ and body weight from baseline. Secondary outcomes were achievement of HbA_1c_ target of ≤48 mmol/mol (≤6.5%) or <53 mmol/mol (<7.0%), body weight loss of at least 10%, and safety outcomes including gastrointestinal adverse events and severe hypoglycaemia. We used version 2 of the Cochrane risk-of-bias tool (ROB 2) to assess the risk of bias, conducted frequentist random-effects network meta-analyses and evaluated confidence in effect estimates utilising the Confidence In Network Meta-Analysis (CINeMA) framework.

**Results:**

A total of 28 trials with 23,622 participants (44.2% female) were included. Compared with placebo, tirzepatide 15 mg was the most efficacious treatment in reducing HbA_1c_ (mean difference −21.61 mmol/mol [−1.96%]) followed by tirzepatide 10 mg (−20.19 mmol/mol [−1.84%]), semaglutide 2.0 mg (−17.74 mmol/mol [−1.59%]), tirzepatide 5 mg (−17.60 mmol/mol [−1.60%]), semaglutide 1.0 mg (−15.25 mmol/mol [−1.39%]) and semaglutide 0.5 mg (−12.00 mmol/mol [−1.09%]). In between-drug comparisons, all tirzepatide doses were comparable with semaglutide 2.0 mg and superior to semaglutide 1.0 mg and 0.5 mg. Compared with placebo, tirzepatide was more efficacious than semaglutide for reducing body weight, with reductions ranging from 9.57 kg (tirzepatide 15 mg) to 5.27 kg (tirzepatide 5 mg). Semaglutide had a less pronounced effect, with reductions ranging from 4.97 kg (semaglutide 2.0 mg) to 2.52 kg (semaglutide 0.5 mg). In between-drug comparisons, tirzepatide 15 mg, 10 mg and 5 mg demonstrated greater efficacy than semaglutide 2.0 mg, 1.0 mg and 0.5 mg, respectively. Both drugs increased incidence of gastrointestinal adverse events compared with placebo, while neither tirzepatide nor semaglutide increased the risk of serious adverse events or severe hypoglycaemia.

**Conclusions/interpretation:**

Our data show that s.c. tirzepatide had a more pronounced effect on HbA_1c_ and weight reduction compared with s.c. semaglutide in people with type 2 diabetes. Both drugs, particularly higher doses of tirzepatide, increased gastrointestinal adverse events.

**Registration:**

PROSPERO registration no. CRD42022382594

**Graphical Abstract:**

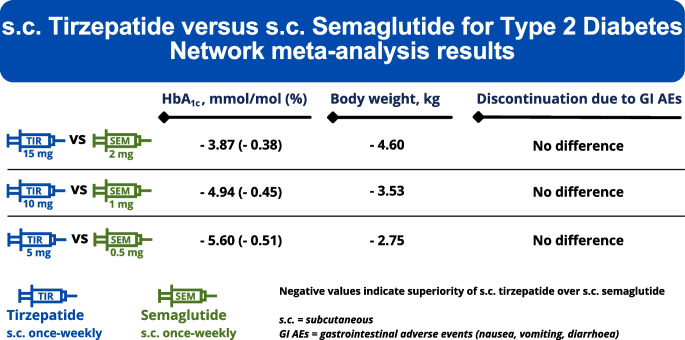

**Supplementary Information:**

The online version contains peer-reviewed but unedited supplementary material available at 10.1007/s00125-024-06144-1.


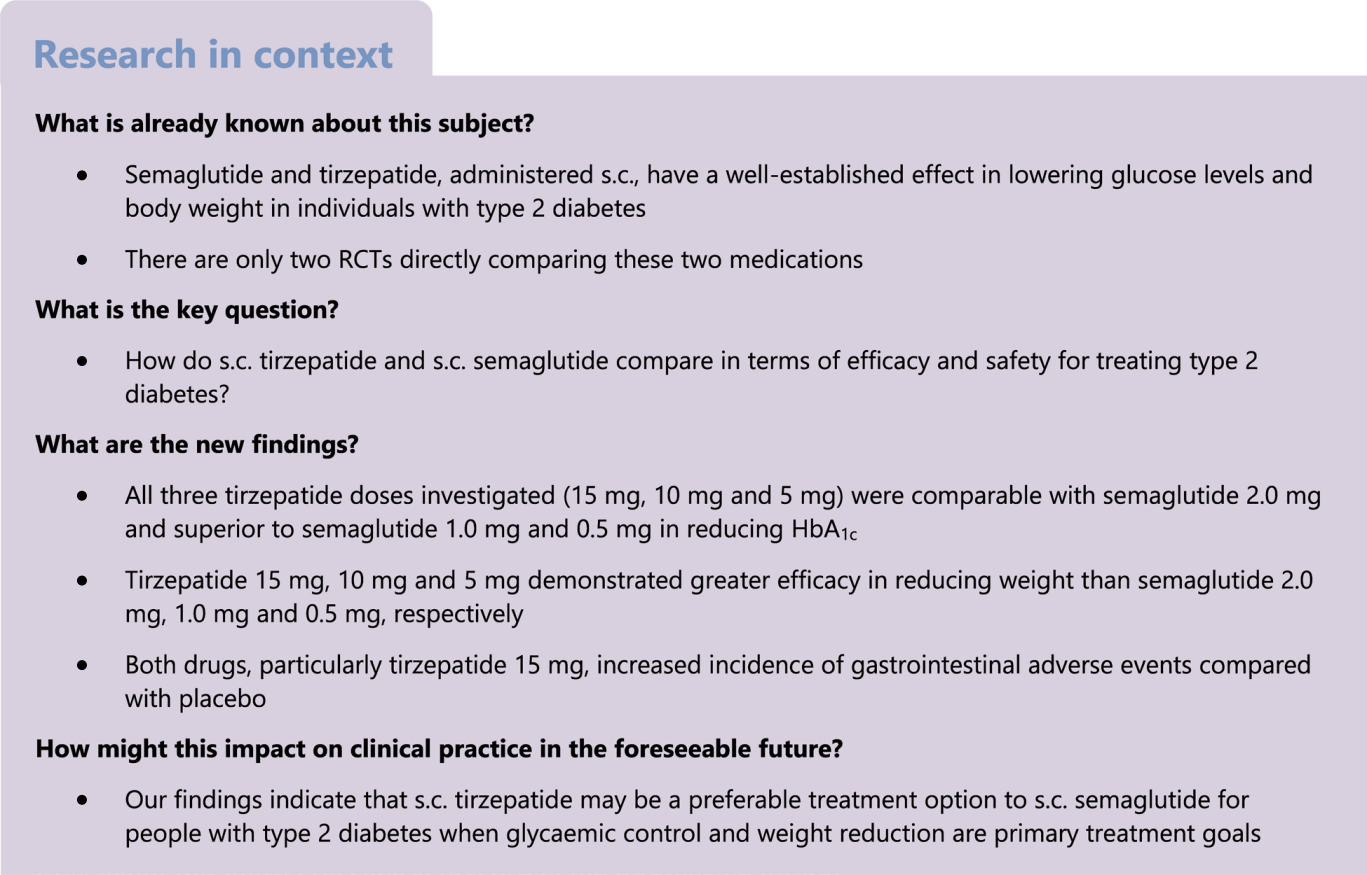


## Introduction

Semaglutide, administered s.c., has shown superior efficacy compared with other glucose-lowering agents, including its oral formulation, in reducing HbA_1c_ and in facilitating weight loss in individuals with type 2 diabetes [1, 2]. Initially approved at doses of 0.5 mg and 1.0 mg once weekly, it has subsequently received authorisation for a 2.0 mg once-weekly dose for the management of type 2 diabetes. Tirzepatide, a novel agent belonging to the glucose-dependent insulinotropic peptide (GIP) and glucagon-like peptide-1 receptor agonist (GLP-1 RA) class (dual GIP/GLP-1 RA), has also been approved by the US Food and Drug Administration (FDA) and the European Medicines Agency (EMA) for the treatment of type 2 diabetes. Data from RCTs have consistently shown the efficacy of tirzepatide in reducing HbA_1c_ and body weight in people with type 2 diabetes [3].

The ADA Standards of Care and the ADA/EASD consensus report recommend s.c. administered semaglutide and tirzepatide as the most efficacious medications for glycaemic control (alongside dulaglutide) and weight reduction [4, 5]. However, direct comparison between s.c. tirzepatide and s.c. semaglutide in RCTs is scarce [6, 7], presenting a challenge in drawing robust and precise conclusions regarding their comparative efficacy. To address this research gap, we conducted a network meta-analysis utilising both direct and indirect comparative data between the two medications [8].

The aim of our systematic review and network meta-analysis was to compare the efficacy (in terms of glycaemic control and weight management) and safety (in terms of adverse events) of s.c. tirzepatide and s.c. semaglutide in people with type 2 diabetes based on data from RCTs.

## Methods

The protocol of this systematic review and meta-analysis is registered in PROSPERO (registration no. CRD42022382594) [9]. We report our methods and results in accordance with the Preferred Reporting Items for Systematic reviews and Meta-Analyses (PRISMA) statement for network meta-analyses [10].

### Eligibility criteria

We included RCTs published in English that assessed s.c. tirzepatide at maintenance doses of 5 mg, 10 mg or 15 mg once weekly, or s.c. semaglutide at maintenance doses of 0.5 mg, 1.0 mg or 2.0 mg once weekly for a minimum duration of 12 weeks. Eligible trials compared any of the specified doses of tirzepatide and semaglutide against each other, placebo or other glucose-lowering drugs. For a glucose-lowering drug to be included as a comparator, it was required to have been evaluated in at least one trial comparison against tirzepatide and one trial comparison against semaglutide. This approach was adopted to prevent unconnected networks, ensuring that each comparator served as a link for indirect comparisons between tirzepatide and semaglutide. We included trials recruiting adults with type 2 diabetes regardless of their background glucose-lowering treatment, defined as the glucose-lowering therapy used both in the intervention and control arms after the randomisation.

### Information sources and searches

We searched PubMed and Cochrane databases from inception until 11 November 2023. Our search strategy included both free-text and Medical Subject Headings (MeSH) terms, utilising the keywords ‘tirzepatide,’ ‘ly3298176,’ ‘semaglutide’ and ‘nn9535’ (electronic supplementary material [ESM] Table 1).

### Study selection

After deduplication, search results were screened at title and abstract level, and potentially eligible records were examined in full text with reasons for exclusion being recorded. Two independent reviewers performed the study selection process and any disagreements were resolved by a third reviewer. For the deduplication and the screening process we used the Systematic Review Accelerator (SRA) web application [11].

### Data collection

Using predesigned forms, we extracted information on study characteristics, participants’ baseline characteristics and outcome data. Given the aggregated data format of the included RCTs in our meta-analysis, direct information on how sex or gender was determined in the individual studies was beyond the scope of our analysis. Our two primary outcomes were the change from baseline in HbA_1c_ and in body weight. Secondary efficacy outcomes were the proportion of participants attaining an HbA_1c_ target of ≤48 mmol/mol (≤6.5%) or <53 mmol/mol (<7.0%), and those achieving a minimum of 10% body weight loss. Safety outcomes included the incidence (no. of participants with at least one outcome event) of nausea, vomiting, diarrhoea, treatment discontinuation due to gastrointestinal events, severe adverse events and severe hypoglycaemia (a hypoglycaemic event requiring assistance). Data were extracted from the intention-to-treat population, which included all randomly assigned participants who received at least one dose of the study medication. For eligible trials identified through our database searches, we utilised ClinicalTrials.gov, using their respective National Clinical Trial (NCT) identifiers, to retrieve additional information when outcome data were absent or incomplete in the published articles. Data extraction was conducted by two independent reviewers, with discrepancies resolved by a third reviewer.

### Risk-of-bias assessment

We used version 2 of the Cochrane risk-of-bias tool for randomised trials (ROB 2) to assess the risk of bias for the two primary outcomes [12]. Following the tool’s algorithms, each trial’s overall risk of bias was classified as low if all domains were at low risk, and high if any domain was at high risk. If none of the domains were classified as high risk but one or more were deemed to have some concerns, the overall risk of bias for that trial was categorised as ‘of some concern’. This assessment was conducted independently by two reviewers, with a third reviewer resolving any disagreements. We evaluated the presence of small-study effect (publication bias) by means of comparison-adjusted funnel plots [13].

### Data analysis

We explored the transitivity assumption by comparing the distribution of potential effect modifiers (baseline HbA_1c_ and body weight) across treatment comparisons [14]. We conducted frequentist random-effects network meta-analyses and calculated mean differences (MDs) for the two primary outcomes and risk ratios for dichotomous outcomes, alongside 95% CIs [15]. We evaluated heterogeneity for the primary outcomes based on the agreement between CIs and prediction intervals in relation to the null effect and the clinically important effect on the opposite direction to the point estimate [16, 17]. We assumed a minimum reduction in HbA_1c_ of 5.5 mmol/mol (0.5%) and in body weight of 4.5 kg (5% of mean body weight value at baseline across all trials) as clinically important [18]. We addressed incoherence (inconsistency) both locally by comparing directly with indirect evidence using the Separating Indirect from Direct Evidence (SIDE) method [19] and globally using the design-by-treatment interaction model [20]. Moreover, we used P-scores, ranging from 0 to 1, to rank treatments; these can be interpreted as the average degree of certainty for a treatment to be better than the other treatments in the network [21]. Statistical analyses were performed in R (R Core Team 2019, R Foundation for Statistical Computing, Vienna, Austria) using the R packages ‘meta’ and ‘netmeta’ [22], and in NMAstudio (version 2.0) web application [23, 24].

### Evaluation of confidence in findings

We evaluated Confidence In Network Meta-Analysis (CINeMA) effect estimates for the primary outcomes utilising the CINeMA methodological framework and application [17, 25]. The six domains evaluated were within-study bias (risk of bias), across-study bias (small-study effect/publication bias), indirectness, imprecision, heterogeneity and incoherence (inconsistency). We assigned judgements at three levels (no concerns, some concerns and major concerns) to each domain and summarised judgements across domains to an overall assessment ranging across very low, low, moderate or high level of confidence [17, 25].

## Results

### Search results and study characteristics

The search retrieved 2798 records, of which 28 RCTs [6, 7, 26–51] with 23,622 participants were included in the systematic review and network meta-analysis (ESM Fig. 1). Study and participant characteristics are presented in Table 1. Only two trials directly compared tirzepatide with semaglutide, with one of these also including a placebo arm [6, 7]. Sixteen trials compared semaglutide with placebo, other GLP-1 RAs, basal insulin, prandial insulin or varying doses of semaglutide. The remaining ten trials compared tirzepatide with placebo, GLP-1 RA (other than semaglutide), basal insulin, prandial insulin or varying doses of tirzepatide. All trials had a parallel-group design and 15 were open-label (Table 1). Most trials were multinational, except for five that recruited exclusively Japanese participants [39–41, 48, 49]. The intervention duration ranged from 24 to 28 weeks in five trials and from 30 to 56 weeks in 21 trials. The remaining two trials, a trial with tirzepatide in people with obesity and type 2 diabetes (SURMOUNT-2) [50] and a cardiovascular outcomes trial with semaglutide (SUSTAIN 6) [31], had a duration of 72 and 104 weeks, respectively. The background glucose-lowering therapy, referring to the common treatment received by all trial groups post-randomisation, varied across the trials. However, the predominant background treatment was metformin, used either as monotherapy or in combination with other medications. Across all trials, 10,442 participants (44.2%) were female, participants’ mean HbA_1c_ at baseline was 66.6 mmol/mol (8.3%), mean body weight was 88.8 kg and mean age was 57.8 years (Table 1). The distribution of potential effect modifiers (HbA_1c_ and body weight at baseline) was deemed sufficiently similar across all treatment comparisons to assume that a network meta-analysis was appropriate (ESM Figs 2 and 3).

**Table 1:** Study details and participant baseline characteristics of included arms in RCTs

Study (trial registration no.)/study arm	Study duration, weeks	Blinding status	Background glucose-lowering therapy^a^	Participants randomised, *n*	Female sex, *n*	Mean HbA_1c_, mmol/mol	Mean HbA_1c_, %	Mean body weight, kg	Mean diabetes duration, years	Mean age, years
SUSTAIN 1 [26] (NCT02054897)	30	Double-blind	None							
Semaglutide 0.5 mg				128	68	64.9	8.1	89.8	4.8	54.6
Semaglutide 1.0 mg				130	50	65.3	8.1	96.9	3.6	52.7
Placebo				129	59	63.4	8.0	89.1	4.1	53.9
SUSTAIN 2 [27] (NCT01930188)	56	Double-blind	Metformin monotherapy (55%) or metformin+TZD (45%)							
Semaglutide 0.5 mg				409	202	64.1	8.0	89.9	6.4	54.8
Semaglutide 1.0 mg				409	204	64.4	8.0	89.2	6.7	56.0
SUSTAIN 3 [28] (NCT01885208)	56	Open-label	Monotherapy or dual combination with metformin (96.5%)/sulfonylurea (48.1%)/TZDs (2.3%)							
Semaglutide 1.0 mg				404	185	67.9	8.4	96.2	9.0	56.4
GLP-1 RA (exenatide extended release)				405	177	67.6	8.3	95.4	9.4	56.7
SUSTAIN 4 [29] (NCT02128932)	30	Open-label	Metformin monotherapy (48%) or metformin+sulfonylurea (52%)							
Semaglutide 0.5 mg				362	165	65.4	8.1	93.7	7.8	56.5
Semaglutide 1.0 mg				360	178	66.6	8.3	94.0	9.3	56.7
Basal insulin (glargine)				360	165	65.4	8.1	92.6	8.6	56.2
SUSTAIN 5 [30] (NCT02305381)	30	Double-blind	Basal insulin monotherapy (16.7%) or basal insulin+metformin (83.3%)							
Semaglutide 0.5 mg				132	58	67.9	8.4	92.7	12.9	59.1
Semaglutide 1 mg				131	54	67.3	8.3	92.5	13.7	58.5
Placebo				133	62	68.6	8.4	89.9	13.3	58.8
SUSTAIN 6 [31] (NCT01720446)	104	Double-blind	None, or monotherapy/dual-combination therapy with any glucose-lowering medication							
Semaglutide 0.5 mg				826	331	71.6	8.7	91.8	14.3	64.6
Semaglutide 1.0 mg				822	304	71.6	8.7	92.9	14.1	64.7
Placebo				1649	660	71.6	8.7	91.9	13.6	64.6
SUSTAIN 7 [32] (NCT02648204)	40	Open-label	Metformin monotherapy							
Semaglutide 0.5 mg				301	132	67.5	8.3	96.4	7.7	56.0
Semaglutide 1.0 mg				300	139	66.2	8.2	95.5	7.3	55.0
GLP-1 RA (dulaglutide)				598	267	65.9	8.2	94.5	7.3	56.0
SUSTAIN 9 [33] (NCT03086330)	30	Double-blind	SGLT2 inhibitor monotherapy (15.6%) or combination of SGLT2 inhibitor with metformin (71.5%)/sulfonylurea (12.9%)							
Semaglutide 1.0 mg				151	62	64.1	8.0	89.6	9.8	57.5
Placebo				151	64	64.5	8.1	93.8	9.6	56.6
SUSTAIN 10 [34] (NCT03191396)	30	Open-label	Metformin monotherapy or any combination of metformin (94.8%)/sulfonylurea (46.8%)/SGLT2 inhibitor (24.6%)							
Semaglutide 1.0 mg				290	130	66.1^b^	8.2	96.6	9.6	60.1
GLP-1 RA (liraglutide)				287	120	67.2^b^	8.3	97.2	8.9	58.9
SUSTAIN 11 [35] (NCT03689374)	52	Open-label	Metformin+insulin glargine							
Semaglutide 1.0 mg				874	429	70.3	8.6	87.6	13.4	60.8
Prandial insulin (aspart)				874	425	69.8	8.5	88.1	13.4	61.5
SUSTAIN CHINA [36] (NCT03061214)	30	Double-blind	Metformin							
Semaglutide 0.5 mg				288	128	65.0^b^	8.1	77.6	6.3	53.0
Semaglutide 1.0 mg				290	136	65.0^b^	8.1	76.1	6.7	53.0
SUSTAIN FORTE [37]** (**NCT03989232)	40	Double-blind	Metformin monotherapy (47%) or metformin+sulfonylurea (53%)							
Semaglutide 1.0 mg				481	197	73.1	8.8	98.6	9.8	58.2
Semaglutide 2.0 mg				480	201	73.4	8.9	100.1	9.2	57.9
Davies et al [38] (NCT01923181)	26	Open-label	None (17.1%) or metformin monotherapy (82.9%)							
Semaglutide 1.0 mg				69	21	61.8^b^	7.8	88.8	5.6	56.8
Placebo				71	31	63.9^b^	8.0	93.8	6.7	58.9
Iijima et al [39] (UMIN000040044)	26	Open-label	None (12.5%), metformin monotherapy (75%), or any combination of metformin/SGLT2 inhibitor/insulin glargine (12.5%)^c^							
Semaglutide 0.5 mg				16	5	46.4	6.4	72.3	13.4	61.5
GLP-1 RA (dulaglutide)				16	1	47.5	6.5	72.7	11.1	62.7
Seino et al [40] (NCT02254291)	30	Open-label	None							
Semaglutide 0.5 mg				103	24	66.1^b^	8.2	67.8	8.0	58.8
Semaglutide 1.0 mg				102	27	63.9^b^	8.0	70.8	7.8	58.1
Takahashi et al [41] (jRCTs1011200008)	24	Open-label	Monotherapy with any glucose-lowering medication or any combination of glucose-lowering medications (87.7% of participants received metformin either as monotherapy or combination therapy)^d^							
Semaglutide 0.5 mg				50	18	63.0	7.9	79.8	NR	62.1
GLP-1 RA (liraglutide or dulaglutide)				50	25	62.0	7.8	78.7	NR	60.9
SURPASS-1 [42] (NCT03954834)	40	Double-blind	None (54%) or previous oral medication use (46%)							
Tirzepatide 5 mg				121	65	63.6	8.0	87.0	4.6	54.1
Tirzepatide 10 mg				121	49	62.9	7.9	86.2	4.9	55.8
Tirzepatide 15 mg				121	58	62.3	7.9	85.4	4.8	52.9
Placebo				115	59	64.5	8.1	84.8	4.5	53.6
SURPASS-2 [6] (NCT03987919)	40	Open-label	Metformin monotherapy							
Tirzepatide 5 mg				470	265	67.5	8.3	92.5	9.1	56.3
Tirzepatide 10 mg				469	231	67.2	8.3	94.8	8.4	57.2
Tirzepatide 15 mg				470	256	66.8	8.3	93.8	8.7	55.9
Semaglutide 1.0 mg				469	244	66.7	8.3	93.7	8.3	56.9
SURPASS-3 [43] (NCT038882970)	52	Open-label	Metformin monotherapy (68%) or metformin+SGLT2 inhibitor (32%)							
Tirzepatide 5 mg				358	158	65.8	8.2	95.4	8.5	57.2
Tirzepatide 10 mg				360	165	66.0	8.2	94.3	8.4	57.4
Tirzepatide 15 mg				358	165	66.3	8.2	94.9	8.5	57.5
Basal insulin (degludec)				359	147	65.4	8.1	94.2	8.1	57.5
SURPASS-4 [44] (NCT03730662)	52	Open-label	Monotherapy with or any combination of metformin (95%)/sulfonylurea (54%)/SGLT2 inhibitor (25%)							
Tirzepatide 5 mg				329	131	69.6	8.5	90.3	9.8	62.9
Tirzepatide 10 mg				328	119	70.4	8.6	90.6	10.6	63.7
Tirzepatide 15 mg				338	135	69.6	8.5	90.0	10.4	63.7
Basal insulin (glargine)				1000	364	69.4	8.5	90.2	10.7	63.8
SURPASS-5 [45] (NCT04039503)	40	Double-blind	Insulin glargine monotherapy (17%) or in combination with metformin (83%)							
Tirzepatide 5 mg				116	55	67.2	8.3	95.8	14.1	62.0
Tirzepatide 10 mg				119	47	67.9	8.4	94.5	12.6	60.0
Tirzepatide 15 mg				120	55	66.5	8.2	96.3	13.7	61.0
Placebo				120	54	68.0	8.4	94.1	12.9	60.0
SURPASS-6 [46] (NCT04537923)	52	Open-label	Metformin+insulin glargine							
Tirzepatide 5 mg				243	144	73.7	8.9	91.7	13.4	58.0
Tirzepatide 10 mg				238	149	72.5	8.8	89.1	13.9	59.6
Tirzepatide 15 mg				236	133	72.0	8.7	91.2	13.4	58.2
Prandial insulin (lispro)				708	396	72.7	8.8	90.3	14.0	59.0
SURPASS-AP-COMBO [47] (NCT04093752)	40	Open-label	Metformin monotherapy (52.5%) or metformin+sulfonylurea (47.5%)							
Tirzepatide 5 mg				230	96	72.4	8.8	77.7	7.4	53.1
Tirzepatide 10 mg				228	102	71.7	8.7	76.3	7.9	53.5
Tirzepatide 15 mg				229	100	71.4	8.7	76.2	7.6	54.3
Basal insulin (glargine)				220	102	71.5	8.7	77.0	7.6	55.6
SURPASS J-COMBO [48] (NCT03861039)	52	Open-label	Monotherapy with sulfonylurea (30%), metformin (14%), α-glucosidase inhibitor (14%), TZD (14%), glinide (14%) or SGLT2 inhibitor (14%)							
Tirzepatide 5 mg				148	29	69.7	8.5	77.7	8.5	57.7
Tirzepatide 10 mg				147	34	70.2	8.6	76.6	9.1	56.9
Tirzepatide 15 mg				148	44	70.0	8.6	78.3	8.5	56.5
SURPASS J-MONO [49] (NCT03861052)	52	Double-blind	None							
Tirzepatide 5 mg				159	46	65.9	8.2	78.5	4.5	56.8
Tirzepatide 10 mg				158	39	66.0	8.2	78.9	5.1	56.2
Tirzepatide 15 mg				160	28	66.1	8.2	78.9	5.1	56.0
GLP-1 RA (dulaglutide)				159	42	65.6	8.2	76.5	5.0	57.5
SURMOUNT-2 [50] (NCT04657003)	72	Double-blind	None or monotherapy with or any combination of metformin (89%), sulfonylurea (27%), SGLT2 inhibitor (20%), TZD (4%), or α-glucosidase inhibitor (1%)							
Tirzepatide 10 mg				312	158	64.0	8.0	100.9	17.6	54.3
Tirzepatide 15 mg				311	159	64.7	8.1	99.6	17.5	53.6
Placebo				315	159	63.7	8.0	101.7	18.1	54.7
Frias et al [51] (NCT03131687)	26	Double-blind	None (9.8%) or metformin monotherapy (90.2%)							
Tirzepatide 5 mg				55	21	66.1	8.2	92.8	8.9	57.9
Tirzepatide 10 mg				51	21	66.1	8.2	92.7	7.9	56.5
Tirzepatide 15 mg				53	31	65.0	8.2	89.1	8.5	56.0
Placebo				51	22	63.9	8.0	91.5	8.6	56.6
GLP-1 RA (dulaglutide)				54	30	65.0	8.1	89.8	9.3	58.7
Heise et al [7] (NCT03951753)	28	Double-blind	Metformin with or without one additional oral medication							
Tirzepatide 15 mg				45	14	62.1	7.8	94.2	10.2	61.1
Semaglutide 1.0 mg				44	10	60.7	7.7	92.7	12.7	63.7
Placebo				28	7	62.9	7.9	98.8	11.0	60.4

^a^Defined as the glucose-lowering treatment received by all trial groups post-randomisation

^b^Data were converted to mmol/mol units from values reported in percentage units using the formula: mmol/mol value = (10.93 × percentage value) − 23.5

^c^All participants received liraglutide before randomisation. After randomisation, participants in the intervention arm switched from liraglutide to semaglutide, while participants in the comparator arm switched from liraglutide to dulaglutide

^d^All participants received GLP-1 RA (liraglutide or dulaglutide) before randomisation. After randomisation, participants in the intervention arm switched to semaglutide, while participants in the comparator continued treatment with liraglutide or dulaglutide

NR, not reported; SGLT2, sodium–glucose cotransporter 2; TZD, thiazolidinedione

### Overview of network

**Fig. 1:**
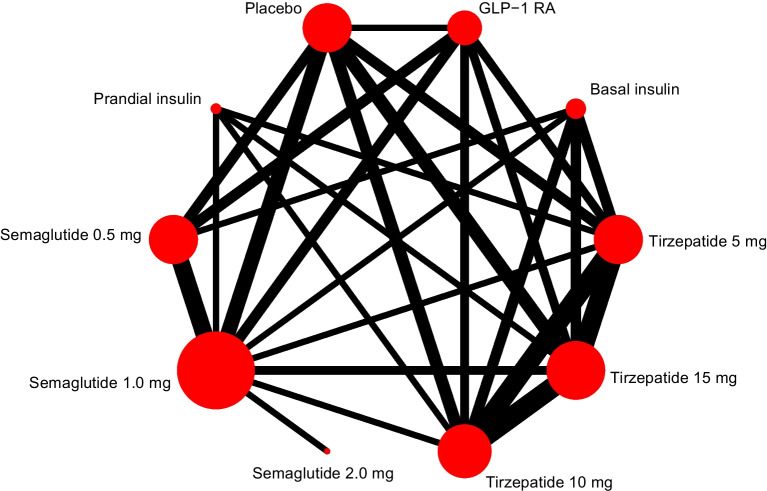
Network plot for change in HbA_1c_. Each circle indicates a treatment node. Lines connecting two nodes represent direct comparisons between two treatments. The size of the nodes is proportional to the number of trials evaluating each treatment; the thickness of the lines is proportional to the number of trials directly comparing the two connected treatments

Figure 1 shows the network of comparisons used in the meta-analysis. Risk of bias for the change in HbA_1c_ was assessed as low in all trials except for one that was at high risk of bias and one with some concerns (ESM Table 2). For the change in body weight, seven trials were at high risk of bias and one trial had some concerns; all other trials were at low risk of bias (ESM Table 3). Comparison-adjusted funnel plots did not suggest the presence of small-study effect (ESM Figs 4 and 5). There was presence of heterogeneity in some comparisons, particularly those involving semaglutide 2.0 mg (ESM Tables 4 and 5). In terms of incoherence, the design-by-treatment interaction model did not identify global inconsistency in the analyses for both primary outcomes (ESM Tables 4 and 5), while local inconsistency was also low.

### Glycaemic efficacy

**Fig. 2:**
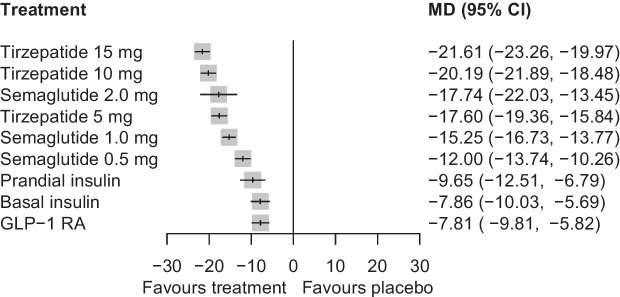
Network meta-analysis results for the change in HbA_1c_ (mmol/mol) compared with placebo

Compared with placebo, tirzepatide 15 mg was the most efficacious treatment in reducing HbA_1c_ (MD [95% CI]: −21.61 mmol/mol [−23.26 to −19.97] [−1.96% (−2.11 to −1.82)]), followed by tirzepatide 10 mg (−20.19 mmol/mol [−21.89 to −18.48] [−1.84% (−1.99 to −1.69)]), semaglutide 2.0 mg (−17.74 mmol/mol [−22.03 to −13.45] [−1.59% (−1.95 to −1.22)]), tirzepatide 5 mg (−17.60 mmol/mol [−19.36 to −15.84] [−1.60% (−1.75 to −1.44)]), semaglutide 1.0 mg (−15.25 mmol/mol [−16.73 to −13.77] [−1.39% (−1.52 to −1.26)]) and semaglutide 0.5 mg (−12.00 mmol/mol [−13.74 to −10.26] [−1.09% (−1.24 to −0.94)]) (Fig. 2 and ESM Fig. 6). In comparisons between tirzepatide and semaglutide, when HbA_1c_ was measured in mmol/mol, all tirzepatide doses were comparable with semaglutide 2.0 mg and superior to semaglutide 1.0 mg and 0.5 mg (ESM Table 6). Specifically, effect estimates (MD [95% CI]) for tirzepatide 15 mg vs semaglutide 2.0 mg, tirzepatide 10 mg vs semaglutide 1.0 mg, and tirzepatide 5 mg vs semaglutide 0.5 mg were, respectively, as follows: −3.87 mmol/mol (−8.22 to 0.48); −4.94 (−6.65 to −3.23); and −5.60 mmol/mol (−7.60 to −3.60) (ESM Table 6). When HbA_1c_ was measured in %, tirzepatide at doses of 15 mg, 10 mg and 5 mg demonstrated greater efficacy than semaglutide at doses of 2.0 mg (MD = −0.38% [95% CI −0.75% to −0.01%]), 1.0 mg (MD = −0.45% [95% CI −0.60% to −0.31%]) and 0.5 mg (MD = −0.51% [95% CI −0.68% to −0.33%]), respectively (ESM Table 7). The confidence in estimates for comparisons between tirzepatide and semaglutide was high to moderate, except for comparisons vs semaglutide 2.0 mg, where the confidence was generally low (ESM Table 8). Consistently with meta-analysis findings, tirzepatide 15 mg held the highest probability (P-score = 0.99) of being the most efficacious treatment in reducing HbA_1c_ (ESM Fig. 7).

Compared with placebo, semaglutide 2.0 mg (risk ratio = 7.73 [95% CI 5.62, 10.63]) and tirzepatide 15 mg (risk ratio = 7.01 [95% CI 5.73, 8.57]) were the most efficacious in achieving an HbA_1c_ target of ≤48 mmol/mol (≤6.5%) (ESM Table 9). In between-drug comparisons, tirzepatide 15 mg and 10 mg outperformed semaglutide 1.0 mg and 0.5 mg and tirzepatide 5 mg was superior to semaglutide 0.5 mg, while no differences were found between semaglutide 2.0 mg and any of the tirzepatide doses (ESM Table 9). Similarly, semaglutide 2.0 mg (risk ratio = 4.01 [95% CI 3.24, 4.95]) and tirzepatide 15 mg (risk ratio = 3.70 [95% CI 3.26, 4.20]) were the most efficacious in achieving an HbA_1c_ target of <53 mmol/mol (<7%) as compared with placebo (ESM Table 10). No differences were found when any of the tirzepatide doses were compared with semaglutide 2.0 mg or 1.0 mg, while all tirzepatide doses were superior to semaglutide 0.5 mg (ESM Table 10).

### Body weight

**Fig. 3:**
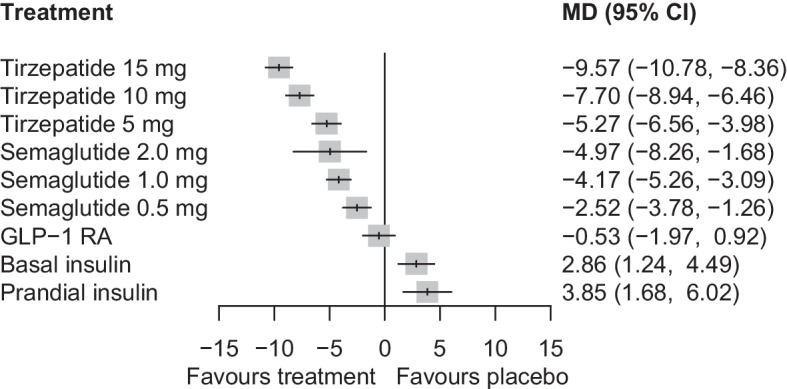
Network meta-analysis results for the change in body weight (kg) compared with placebo

In comparisons vs placebo, tirzepatide was the most efficacious medication for lowering body weight, resulting in reductions ranging from 9.57 kg (95% CI 8.36, 10.78) with tirzepatide 15 mg to 5.27 kg (95% CI 3.98, 6.56) with tirzepatide 5 mg (Fig. 3). Semaglutide showed a less pronounced effect, with reductions ranging from 4.97 kg (95% CI 1.68, 8.26) with semaglutide 2.0 mg to 2.52 kg (95% CI 1.26, 3.78) with semaglutide 0.5 mg (Fig. 3). In between-drug comparisons, tirzepatide at doses of 15 mg, 10 mg and 5 mg demonstrated greater efficacy than semaglutide at doses of 2.0 mg (MD = −4.60 kg [95% CI −7.94, −1.26]), 1.0 mg (MD = −3.53 kg [95% CI −4.80, −2.25]) and 0.5 mg (MD = −2.75 kg [95% CI −4.23, −1.28]), respectively (ESM Table 11). The confidence in estimates for comparisons between tirzepatide and semaglutide was high to moderate, except for comparisons vs semaglutide 2.0 mg, where the confidence was low (ESM Table 12). Tirzepatide 15 mg was ranked highest (P-score = 1.00) among all treatments in terms of weight reduction (ESM Fig. 8).

All doses of tirzepatide and semaglutide were superior to placebo in achieving at least a 10% body weight reduction, with tirzepatide 15 mg (risk ratio = 10.51 [95% CI 7.55, 14.64]) and tirzepatide 10 mg (risk ratio = 8.84 [95% CI 6.35, 12.32]) being the most efficacious treatments (ESM Table 13). In between-drug comparisons, tirzepatide at both the 15 mg and 10 mg doses outperformed all doses of semaglutide, while tirzepatide at the 5 mg dose was more efficacious than semaglutide 0.5 mg (ESM Table 13).

### Gastrointestinal adverse events

**Fig. 4:**
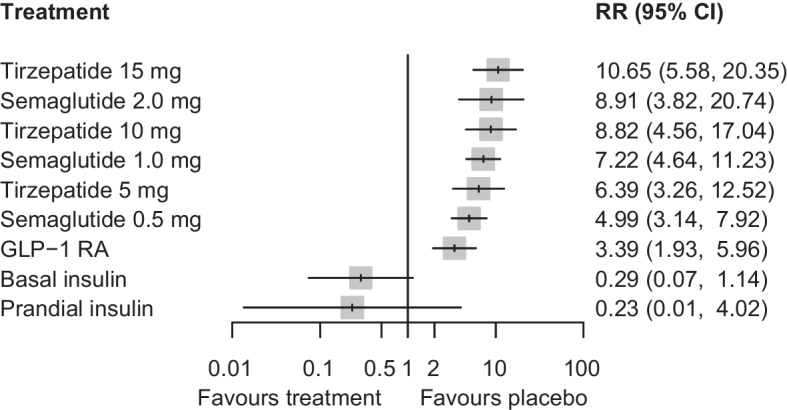
Network meta-analysis results for the discontinuation of treatment due to gastrointestinal adverse events compared with placebo. RR, risk ratio

Compared with placebo, all doses of tirzepatide and semaglutide demonstrated an increase in the risk for nausea (ESM Fig. 9), vomiting (ESM Fig. 10) and diarrhoea (ESM Fig. 11). Specifically, the risk ratios for nausea ranged from 2.07 to 3.51 across different doses of tirzepatide, and from 2.45 to 2.84 for semaglutide (ESM Table 14). For vomiting, the risk ratios ranged from 2.39 to 4.36 with tirzepatide, and from 2.33 to 3.62 with semaglutide (ESM Table 15). For diarrhoea, the risk ratios ranged from 1.81 to 2.18 with tirzepatide, and from 1.66 to 1.80 with semaglutide (ESM Table 16). In comparisons between tirzepatide and semaglutide, all doses of tirzepatide had similar risk profiles for gastrointestinal adverse events when compared with semaglutide 2.0 mg. However, tirzepatide 15 mg and 10 mg generally exhibited an increased risk compared with semaglutide 1.0 mg and 0.5 mg (ESM Tables 14–16). Discontinuation of treatment due to gastrointestinal adverse events was more frequent with any dose of tirzepatide (risk ratios ranging from 6.39 to 10.65) or semaglutide (risk ratios ranging from 4.99 to 8.91) compared with placebo (Fig. 4). No differences were observed when comparing tirzepatide with semaglutide, except for tirzepatide 15 mg vs semaglutide 0.5 mg (ESM Table 17).

### Serious adverse events and severe hypoglycaemia

Neither tirzepatide nor semaglutide were associated with an increased risk for serious adverse events when compared with placebo (ESM Fig. 12), and no differences were observed in the comparisons between tirzepatide and semaglutide (ESM Table 18). We did not conduct a meta-analysis for severe hypoglycaemia due to the absence of events in most treatment arms across all trials. In particular, in the overall population, 107 participants experienced an episode of severe hypoglycaemia, with 30 of the cases occurring in a single trial arm wherein participants were randomised to prandial insulin [46].

## Discussion

Our systematic review and network meta-analysis provides an up-to-date evidence synthesis on the comparative efficacy of the FDA- and EMA-approved doses of s.c. semaglutide and tirzepatide for type 2 diabetes. All tirzepatide doses were comparable with semaglutide 2.0 mg and superior to semaglutide 1.0 mg and 0.5 mg in reducing HbA_1c_. In terms of body weight reduction, tirzepatide at doses of 15 mg, 10 mg and 5 mg demonstrated greater efficacy than semaglutide at doses of 2.0 mg, 1.0 mg and 0.5 mg, respectively. All doses of both drugs, particularly tirzepatide 15 mg, increased the occurrence of gastrointestinal adverse events vs placebo. Neither tirzepatide nor semaglutide increased the risk for serious adverse events or severe hypoglycaemia.

Unlike a previous network meta-analysis, which was limited to eight RCTs featuring only tirzepatide [52], and another focusing exclusively on semaglutide [53], we compared the two medications by including RCTs that either directly compared s.c. tirzepatide with s.c. semaglutide or used any common comparator such as placebo, basal insulin, prandial insulin or another GLP-1 RA. Moreover, our systematic review extends beyond the scope of another recent network meta-analysis, which, while including s.c. tirzepatide and high-dose GLP-1 RA, did not account for lower, yet clinically relevant, s.c. doses of semaglutide (1.0 mg and 0.5 mg) [54]. Conversely, we included these doses and incorporated data from two additional recent RCTs with tirzepatide [46, 50]. Furthermore, our systematic review builds upon the findings of a network meta-analysis that found s.c. tirzepatide to be more effective than s.c. semaglutide in weight management [55]. However, the authors included only six RCTs involving tirzepatide and, as opposed to our meta-analysis, did not provide information on comparative effects across different doses of the two medications [55]. Our network meta-analysis, while reaching similar conclusions to another recent network meta-analysis regarding the more pronounced effect of s.c. tirzepatide over s.c. semaglutide in reducing HbA_1c_ and body weight [56], differs in methodology and scope. In particular, as opposed to Ding et al’s Bayesian approach [56], we employed a frequentist method and were more selective in our inclusion criteria, focusing on the s.c. formulation of semaglutide due to its demonstrated efficacy over other glucose-lowering agents, including orally administered semaglutide [1, 2]. Furthermore, our analysis incorporated six additional RCTs, including a trial with s.c. semaglutide 2.0 mg and two recently published trials with s.c. tirzepatide [7, 37, 41, 46, 48, 50]. In addition, we focused on comparisons of clinically approved doses for both drugs, omitting lower doses that are not used in clinical practice, and formally evaluated the confidence in meta-analysis findings [25]. Finally, our findings offer a more comprehensive assessment compared with another analysis that produced indirect estimates between tirzepatide and semaglutide 2.0 mg using data solely from two RCTs [57]. As opposed to this study, we did not find a difference between tirzepatide 10 mg and semaglutide 2.0 mg in terms of HbA_1c_ or body weight reduction. These differences likely arise from our much larger dataset encompassing 28 RCTs, allowing for more accurate comparative estimates between treatments.

Specific limitations should be acknowledged. Given that our systematic review was designed to assess the comparative efficacy and safety between s.c. tirzepatide and s.c. semaglutide, eligible RCTs focused on either direct comparisons between these two medications or vs common comparators (comparators that have been assessed in at least one trial comparison against s.c. tirzepatide and in one trial comparison against s.c. semaglutide). This focused approach, while providing insights into comparisons between tirzepatide and semaglutide, is not as well suited for an evaluation of the two medications vs the common comparators included in the analysis. Moreover, we observed low confidence in meta-analysis results in comparisons involving semaglutide 2.0 mg, attributable to the inclusion of only one RCT assessing this dose. As such, interpretations concerning the comparative efficacy and safety of semaglutide 2.0 mg vs tirzepatide doses should be approached with caution. In addition, the treatment response observed in our analysis may also be influenced by ethnic differences, given that five RCTs recruited exclusively Japanese participants [39–41, 48, 49]. In particular, it has been shown that East Asian people with type 2 diabetes typically present with less severe obesity and are characterised by lower beta cell function and lesser insulin resistance compared with White populations [58]. Furthermore, evidence suggests that sex differences may influence the efficacy and safety profiles of GLP-1 RAs [59]. Specifically, women treated with GLP-1 RAs may experience greater glycaemic control and weight-reduction benefits, as well as a higher incidence of gastrointestinal adverse events, compared with men [59]. However, we did not perform subgroup analyses based on sex. This limitation reflects the broader issue of inconsistent reporting of sex-disaggregated outcomes in diabetes-treatment research and underscores the need for future research to systematically explore and report the effects of diabetes treatments according to sex. Another limitation is the a priori exclusion of long-term cardiovascular or mortality outcomes from our analysis, a decision based on the fact that the dedicated cardiovascular outcomes trial for tirzepatide (SURPASS-CVOT) is still ongoing [60]. Finally, in our analysis of the change in HbA_1c_, we noted a variation in the results based on the measurement units used. Specifically, when HbA_1c_ was measured in mmol/mol, semaglutide 2.0 mg showed a marginally more pronounced effect compared with placebo than tirzepatide 5 mg, whereas this trend was reversed when analysing HbA_1c_ in percentage units. Of note, the percentage-based results are potentially more precise, as they did not require imputations or borrowing of variance values from other studies. This aspect was necessary in the mmol/mol analysis due to some studies not reporting complete measures of variance, highlighting the need for future trials to report results for HbA_1c_ in both mmol/mol and percentage units to aid in comprehensive analysis and interpretation of findings.

There were some protocol deviations in our analysis. We did not include achievement of at least a 5% reduction in body weight as an outcome because all doses of both drugs were superior to placebo in achieving at least a 10% weight reduction. As such, including an additional outcome with less clinically meaningful threshold would overload the study results with redundant information rather than providing added clinical insights. Our plan to conduct sensitivity or subgroup analyses based on risk-of-bias assessment, trial duration and background glucose-lowering treatment was also not implemented. The sensitivity analysis based on risk of bias was not performed because only a few trials were at high risk of bias, while the average risk of bias across treatment comparisons was incorporated into our assessment of confidence in the findings using the CINeMA framework [25]. Regarding trial duration, this ranged between 26 weeks and 56 weeks in all trials except for two, making a subgroup analysis based on duration unwarranted. Furthermore, performing subgroup analyses based on background glucose-lowering therapy was not feasible due to the varied treatments across trials. However, in most trials, participants received metformin, either as monotherapy or in combination with other agents, except for three trials where participants received no background glucose-lowering therapy after randomisation [26, 40, 49].

Clinical practice recommendations by the ADA Standards of Care and the ADA/EASD consensus report place s.c. tirzepatide and s.c. semaglutide among the most efficacious treatment for lowering glucose (alongside dulaglutide) and reducing weight in people with type 2 diabetes [4, 5]. In line with these recommendations, our meta-analysis corroborates the clinical benefits of both medications compared with placebo. Our findings also suggest that s.c. tirzepatide could be a preferable option over s.c. semaglutide for individuals who prioritise glycaemic and weight management due to its more pronounced effect in both outcomes. However, the goal of glucose-lowering therapy extends beyond controlling blood glucose levels and body weight, encompassing the reduction of long-term cardiovascular complications. For example, s.c. semaglutide has demonstrated cardiovascular benefits in the SUSTAIN-6 trial, which was designed to assess the non-inferiority of s.c. semaglutide as compared with placebo in terms of cardiovascular safety in people with type 2 diabetes at increased cardiovascular risk [31]. While pooled data from the SURPASS clinical trial programme indicate that tirzepatide does not increase the risk of major cardiovascular events [61], definitive conclusions regarding its cardiovascular profile should await the results of SURPASS-CVOT, with its completion anticipated in late 2024 [60]. Furthermore, our analysis suggests that the increased gastrointestinal adverse events associated with both s.c. tirzepatide and s.c. semaglutide can lead to treatment discontinuation in some patients, particularly with the higher dose of tirzepatide. In older and frail individuals, where vomiting and diarrhoea could result in dehydration, these medications might need to be prescribed with caution.

Complementing our meta-analysis findings, a recent preprint of a large observational study comparing s.c. tirzepatide with s.c. semaglutide in the USA provides valuable real-world evidence [62]. This study found that s.c. tirzepatide was more effective than s.c. semaglutide in reducing body weight among obese or overweight individuals, a benefit that was apparent regardless of the presence of type 2 diabetes, while the rates of gastrointestinal adverse events were similar between the two drugs [62]. These real-world findings provide insights into the effectiveness and tolerability of these medications outside the controlled environment of RCTs, reinforcing the potential of tirzepatide as a highly effective option for weight management in routine clinical practice. However, it is essential to consider the broader implications of adopting these therapies in real-world settings, particularly concerning their cost. Observational data suggest a notable under-utilisation of GLP-1 RAs among individuals in lower socioeconomic groups, primarily due to the high cost of these medications, highlighting the disparity in access to effective diabetes treatments based on socioeconomic factors [63]. From a broader societal perspective, even though s.c. tirzepatide has been suggested to be cost-effective compared with s.c. semaglutide in the USA [64], cost-effectiveness analyses conducted in low-, middle- and high-income countries have demonstrated that GLP-1 RAs are not cost-effective compared with other glucose-lowering drugs [63].

### Conclusions

Our network meta-analysis of 28 RCTs found that s.c. tirzepatide generally had a more pronounced effect than s.c. semaglutide in reducing HbA_1c_ and body weight in people with type 2 diabetes. Notably, both drugs, particularly the higher doses of tirzepatide, were associated with an increased incidence of gastrointestinal adverse events. These findings can inform clinical decisions and optimising treatment strategies in the management of type 2 diabetes.

### Supplementary Information

Below is the link to the electronic supplementary material.

Supplementary file1 (PDF 2326 KB)

## Data Availability

The datasets generated and/or analysed during the current study are available from the corresponding author on reasonable request.

## Abbreviations

CINeMA: Confidence In Network Meta-Analysis
EMA: European Medicines Agency
FDA: US Food and Drug Administration
GIP: Glucose-dependent insulinotropic peptide
GLP-1 RA: Glucagon-like peptide-1 receptor agonist
MD: Mean difference
