# Supplementary material for: Subcutaneously administered tirzepatide vs semaglutide for adults with type 2 diabetes: a systematic review and network meta-analysis of randomised controlled trials
Source: Diabetologia. 2024 Apr 13;67(7):1206–22. doi: 10.1007/s00125-024-06144-1 (PMC11153294; doi:10.1007/s00125-024-06144-1)

Electronic Supplementary Material (ESM)

ESM Tables 3

ESM Table 1 Search strategy 3

ESM Table 2 Risk of bias assessment of included trials for the change from baseline in HbA1c (mmol/mol) 4

ESM Table 3 Risk of bias assessment of included trials for the change from baseline in body weight (kg) 5

ESM Table 4 Evaluation of heterogeneity (prediction intervals) and inconsistency (incoherence) in the network meta-analysis for the change from baseline in HbA1c (mmol/mol) 6

ESM Table 5 Evaluation of heterogeneity (prediction intervals) and inconsistency (incoherence) in the network meta-analysis for the change from baseline in body weight (kg) 7

ESM Table 6 Network meta-analysis results for the change from baseline in HbA1c (mmol/mol) 8

ESM Table 7 Network meta-analysis results for the change from baseline in HbA1c (%) 9

ESM Table 8 Confidence in effect estimates for the change from baseline in HbA1c (mmol/mol) in comparisons between tirzepatide, semaglutide, and placebo 10

ESM Table 9 Network meta-analysis results for achievement of an HbA1c target of ≤48 mmol/mol (≤6.5%) 11

ESM Table 10 Network meta-analysis results for achievement of an HbA1c target of <53 mmol/mol (<7.0%) 12

ESM Table 11 Network meta-analysis results for the change from baseline in body weight (kg) 13

ESM Table 12 Confidence in effect estimates for the change from baseline in in body weight (kg) in comparisons between tirzepatide to semaglutide to and placebo 14

ESM Table 13 Network meta-analysis results for achievement of at least a 10% body weight reduction 15

ESM Table 14 Network meta-analysis results for the incidence of nausea 16

ESM Table 15 Network meta-analysis results for the incidence of vomiting 17

ESM Table 16 Network meta-analysis results for the incidence of diarrhoea 18

ESM Table 17 Network meta-analysis results for treatment discontinuation due to gastrointestinal adverse events 19

ESM Table 18 Network meta-analysis results for the incidence of serious adverse events 20

ESM Figures 21

ESM Fig. 1 PRISMA flow diagram of the study selection process 21

ESM Fig. 2 Assessment of transitivity based on the distribution of mean baseline HbA1c (%) 22

ESM Fig. 3 Assessment of transitivity based on the distribution of mean baseline body weight (kg) .. 23

ESM Fig. 4 Comparison-adjusted funnel plot for the change from baseline in HbA1c (mmol/mol) 24

ESM Fig. 5 Comparison-adjusted funnel plot for the change from baseline in body weight (kg) 25

ESM Fig. 6 Network meta-analysis results compared to placebo for the change from baseline in HbA1c (%). MD, mean difference 26

ESM Fig. 7 Treatment ranking (P-scores) for the change from baseline in HbA1c (mmol/mol). P-scores range from 0 to 1 to and can be interpreted as an average degree of probability for a treatment to be better than the other treatments in the network 27

ESM Fig. 8 Treatment ranking (P-scores) for the change from baseline in body weight (kg). P-scores range from 0 to 1 to and can be interpreted as an average degree of probability for a treatment to be better than the other treatments in the network 28

ESM Fig. 9 Network meta-analysis results compared to placebo for the incidence of nausea. RR, risk ratio 29

ESM Fig. 10 Network meta-analysis results compared to placebo for the incidence of vomiting. RR, risk ratio 30

ESM Fig. 11 Network meta-analysis results compared to placebo for the incidence of diarrhoea. RR, risk ratio 31

ESM Fig. 12 Network meta-analysis results compared to placebo for the incidence of serious adverse events. RR, risk ratio 32

ESM Tables
ESM Table 1 Search strategy

PubMed:

1. tirzepatide
2. ly3298176
3. ly-3298176
4. semaglutide
5. nn9535
6. nn-9535
7. OR 1-6

Cochrane Library:

1. (tirzepatide)
2. (ly3298176)
3. (ly-3298176)
4. (semaglutide)
5. (nn9535)
6. (nn-9535)
7. OR 1-6

ESM Table 2 Risk of bias assessment of included trials for the change from baseline in HbA1c (mmol/mol)

Study	Risk of bias domains					
	**Randomisation process**	**Deviations from the intended interventions**	**Missing outcome data**	**Measurement of the outcome**	**Selection of the reported result**	**Overall assessment**
SUSTAIN 3 [28]	L	L	L	L	L	L
SUSTAIN 4 [29]	L	L	L	L	L	L
SUSTAIN 10 [34]	L	L	L	L	L	L
Davies 2017 [38]	L	H	L	L	L	H
SUSTAIN FORTE [37]	L	L	L	L	L	L
SUSTAIN 6 [31]	L	L	L	L	L	L
SUSTAIN 7 [32]	L	L	L	L	L	L
SUSTAIN 5 [30]	L	L	L	L	L	L
SUSTAIN 1 [26]	L	L	L	L	L	L
SUSTAIN 9 [33]	L	L	L	L	L	L
SUSTAIN 2 [27]	L	L	L	L	L	L
SUSTAIN China [36]	L	L	L	L	L	L
SUSTAIN 11 [35]	L	L	L	L	L	L
Seino 2017 [40]	L	L	L	L	L	L
Iijima 2023 [39]	S	S	L	L	L	S
Takahashi 2023 [41]	L	L	L	L	L	L
SURPASS-5 [45]	L	L	L	L	L	L
SURPASS-4 [44]	L	L	L	L	L	L
Frias 2018 [51]	L	L	L	L	L	L
SURPASS-2 [6]	L	L	L	L	L	L
Heise 2022 [7]	L	L	L	L	L	L
SURPASS J-MONO [49]	L	L	L	L	L	L
SURPASS J-COMBO [48]	L	L	L	L	L	L
SURPASS-3 [43]	L	L	L	L	L	L
SURPASS-1 [42]	L	L	L	L	L	L
SURPASS-AP-COMBO [47]	L	L	L	L	L	L
SURMOUNT-2 [50]	L	L	L	L	L	L
SURPASS-6 [46]	L	L	L	L	L	L
H, high risk of bias; L, low risk of bias; S, some concerns						

ESM Table 3 Risk of bias assessment of included trials for the change from baseline in body weight (kg)

**Study**	**Risk of bias domains**					
	**Randomisation process**	**Deviations from the intended interventions**	**Missing outcome data**	**Measurement of the outcome**	**Selection of the reported result**	**Overall assessment**
SUSTAIN 3 [28]	L	L	L	L	L	L
SUSTAIN 4 [29]	L	H	L	L	L	H
SUSTAIN 10 [34]	L	L	L	L	L	L
Davies 2017 [38]	L	H	L	L	L	H
SUSTAIN FORTE [37]	L	L	L	L	L	L
SUSTAIN 6 [31]	L	L	L	L	L	L
SUSTAIN 7 [32]	L	L	L	L	L	L
SUSTAIN 5 [30]	L	L	L	L	L	L
SUSTAIN 1 [26]	L	L	L	L	L	L
SUSTAIN 9 [33]	L	L	L	L	L	L
SUSTAIN 2 [27]	L	L	L	L	L	L
SUSTAIN China [36]	L	L	L	L	L	L
SUSTAIN 11 [35]	L	H	L	L	L	H
Seino 2017 [40]	L	L	L	L	L	L
Iijima 2023 [39]	S	S	L	L	L	S
Takahashi 2023 [41]	L	L	L	L	L	L
SURPASS-5 [45]	L	L	L	L	L	L
SURPASS-4 [44]	L	H	L	L	L	H
Frias 2018 [51]	L	L	L	L	L	L
SURPASS-2 [6]	L	L	L	L	L	L
Heise 2022 [7]	L	L	L	L	L	L
SURPASS J-MONO [49]	L	L	L	L	L	L
SURPASS J-COMBO [48]	L	L	L	L	L	L
SURPASS-3 [43]	L	H	L	L	L	H
SURPASS-1 [42]	L	L	L	L	L	L
SURPASS-AP-COMBO [47]	L	H	L	L	L	H
SURMOUNT-2 [50]	L	L	L	L	L	L
SURPASS-6 [46]	L	H	L	L	L	H
H, high risk of bias; L, low risk of bias; S, some concerns						

ESM Table 4 Evaluation of heterogeneity (prediction intervals) and inconsistency (incoherence) in the network meta-analysis for the change from baseline in HbA1c (mmol/mol)

**Heterogeneity**	
**Comparison**	**Prediction interval (lower limit, upper limit)**
Placebo:Semaglutide 0.5 mg	(7.755, 16.247)
Placebo:Semaglutide 1.0 mg	(11.110, 19.386)
Placebo:Tirzepatide 10 mg	(15.958, 24.414)
Placebo:Tirzepatide 15 mg	(17.408, 25.816)
Placebo:Tirzepatide 5 mg	(13.349, 21.853)
Semaglutide 0.5 mg:Semaglutide 1.0 mg	(-0.856, 7.350)
Semaglutide 1.0 mg:Semaglutide 2.0 mg	(-3.161, 8.141)
Semaglutide 1.0 mg:Tirzepatide 10 mg	(0.707, 9.168)
Semaglutide 1.0 mg:Tirzepatide 15 mg	(2.158, 10.569)
Semaglutide 1.0 mg:Tirzepatide 5 mg	(-1.892, 6.599)
Tirzepatide 10 mg:Tirzepatide 15 mg	(-2.653, 5.505)
Tirzepatide 10 mg:Tirzepatide 5 mg	(-6.684, 1.515)
Tirzepatide 15 mg:Tirzepatide 5 mg	(-8.105, 0.084)
Placebo:Semaglutide 2.0 mg	(11.886, 23.590)
Semaglutide 0.5 mg:Semaglutide 2.0 mg	(-0.090, 11.564)
Semaglutide 0.5 mg:Tirzepatide 10 mg	(3.835, 12.534)
Semaglutide 0.5 mg:Tirzepatide 15 mg	(5.283, 13.938)
Semaglutide 0.5 mg:Tirzepatide 5 mg	(1.236, 9.964)
Semaglutide 2.0 mg:Tirzepatide 10 mg	(-3.470, 8.365)
Semaglutide 2.0 mg:Tirzepatide 15 mg	(-2.026, 9.773)
Semaglutide 2.0 mg:Tirzepatide 5 mg	(-6.065, 5.792)

**Inconsistency (design-by-treatment model)**	
Q:	19.430
Degrees of freedom:	28
P value:	0.884

ESM Table 5 Evaluation of heterogeneity (prediction intervals) and inconsistency (incoherence) in the network meta-analysis for the change from baseline in body weight (kg)

**Heterogeneity**	
**Comparison**	**Prediction interval (lower limit, upper limit)**
Placebo:Semaglutide 0.5 mg	(-0.829, 5.863)
Placebo:Semaglutide 1.0 mg	(0.890, 7.453)
Placebo:Tirzepatide 10 mg	(4.360, 11.036)
Placebo:Tirzepatide 15 mg	(6.244, 12.897)
Placebo:Tirzepatide 5 mg	(1.914, 8.629)
Semaglutide 0.5 mg:Semaglutide 1.0 mg	(-1.599, 4.909)
Semaglutide 1.0 mg:Semaglutide 2.0 mg	(-3.637, 5.237)
Semaglutide 1.0 mg:Tirzepatide 10 mg	(0.172, 6.880)
Semaglutide 1.0 mg:Tirzepatide 15 mg	(2.058, 8.739)
Semaglutide 1.0 mg:Tirzepatide 5 mg	(-2.266, 4.465)
Tirzepatide 10 mg:Tirzepatide 15 mg	(-1.371, 5.116)
Tirzepatide 10 mg:Tirzepatide 5 mg	(-5.685, 0.832)
Tirzepatide 15 mg:Tirzepatide 5 mg	(-7.554, -1.043)
Placebo:Semaglutide 2.0 mg	(0.397, 9.547)
Semaglutide 0.5 mg:Semaglutide 2.0 mg	(-2.100, 7.010)
Semaglutide 0.5 mg:Tirzepatide 10 mg	(1.756, 8.606)
Semaglutide 0.5 mg:Tirzepatide 15 mg	(3.640, 10.467)
Semaglutide 0.5 mg:Tirzepatide 5 mg	(-0.682, 6.192)
Semaglutide 2.0 mg:Tirzepatide 10 mg	(-1.901, 7.354)
Semaglutide 2.0 mg:Tirzepatide 15 mg	(-0.019, 9.216)
Semaglutide 2.0 mg:Tirzepatide 5 mg	(-4.336, 4.936)

**Inconsistency (design-by-treatment model)**	
Q:	33.464
Degrees of freedom:	28
P value:	0.219

ESM Table 6 Network meta-analysis results for the change from baseline in HbA1c (mmol/mol)

**Tirzepatide 15 mg**									
-1.43 (-2.74 to -0.11)	**Tirzepatide 10 mg**								
-3.87 (-8.22 to 0.48)	-2.45 (-6.82 to 1.93)	**Semaglutide 2.0 mg**							
-4.01 (-5.37 to -2.65)	-2.58 (-3.96 to -1.21)	-0.14 (-4.52 to 4.25)	**Tirzepatide 5 mg**						
-6.36 (-8.01 to -4.72)	-4.94 (-6.65 to -3.23)	-2.49 (-6.52 to 1.54)	-2.35 (-4.10 to -0.61)	**Semaglutide 1.0 mg**					
-9.61 (-11.54 to -7.69)	-8.18 (-10.16 to -6.21)	-5.74 (-9.99 to -1.48)	-5.60 (-7.60 to -3.60)	-3.25 (-4.63 to -1.86)	**Semaglutide 0.5 mg**				
-11.96 (-14.69 to -9.23)	-10.53 (-13.28 to -7.79)	-8.09 (-12.93 to -3.24)	-7.95 (-10.71 to -5.19)	-5.60 (-8.29 to -2.91)	-2.35 (-5.28 to 0.59)	**Prandial insulin**			
-13.75 (-15.63 to -11.87)	-12.32 (-14.22 to -10.43)	-9.88 (-14.41 to -5.35)	-9.74 (-11.65 to -7.83)	-7.39 (-9.47 to -5.31)	-4.14 (-6.39 to -1.89)	-1.79 (-4.87 to 1.29)	**Basal insulin**		
-13.80 (-15.79 to -11.80)	-12.37 (-14.40 to -10.34)	-9.92 (-14.29 to -5.56)	-9.79 (-11.84 to -7.74)	-7.43 (-9.12 to -5.75)	-4.19 (-6.01 to -2.37)	-1.84 (-4.86 to 1.19)	-0.05 (-2.44 to 2.35)	**GLP-1 RA**	
-21.61 (-23.26 to -19.97)	-20.19 (-21.89 to -18.48)	-17.74 (-22.03 to -13.45)	-17.60 (-19.36 to -15.84)	-15.25 (-16.73 to -13.77)	-12.00 (-13.74 to -10.26)	-9.65 (-12.51 to -6.79)	-7.86 (-10.03 to -5.69)	-7.81 (-9.81 to -5.82)	**Placebo**
Treatment estimates are mean differences (MDs) and 95% CIs in the column-defining treatment compared to the row-defining treatment. Values lower than 0 favour the column-defining treatment.									

ESM Table 7 Network meta-analysis results for the change from baseline in HbA1c (%)

**Tirzepatide 15 mg**									
-0.12 (-0.23 to -0.01)	**Tirzepatide 10 mg**								
-0.37 (-0.49 to -0.25)	-0.25 (-0.37 to -0.13)	**Tirzepatide 5 mg**							
-0.38 (-0.75 to -0.01)	-0.25 (-0.63 to 0.12)	-0.01 (-0.38 to 0.37)	**Semaglutide 2.0 mg**						
-0.58 (-0.72 to -0.43)	-0.45 (-0.60 to -0.31)	-0.21 (-0.36 to -0.06)	-0.20 (-0.54 to 0.14)	**Semaglutide 1.0 mg**					
-0.88 (-1.04 to -0.71)	-0.76 (-0.93 to -0.58)	-0.51 (-0.68 to -0.33)	-0.50 (-0.86 to -0.14)	-0.30 (-0.42 to -0.18)	**Semaglutide 0.5 mg**				
-1.03 (-1.26 to -0.80)	-0.91 (-1.14 to -0.68)	-0.66 (-0.89 to -0.43)	-0.66 (-1.06 to -0.25)	-0.46 (-0.68 to -0.23)	-0.15 (-0.40 to 0.09)	**Prandial insulin**			
-1.23 (-1.41 to -1.05)	-1.11 (-1.29 to -0.93)	-0.86 (-1.05 to -0.68)	-0.86 (-1.23 to -0.48)	-0.66 (-0.80 to -0.51)	-0.35 (-0.51 to -0.19)	-0.20 (-0.46 to 0.06)	**GLP-1 RA**		
-1.25 (-1.41 to -1.09)	-1.13 (-1.29 to -0.96)	-0.88 (-1.04 to -0.72)	-0.87 (-1.26 to -0.49)	-0.67 (-0.85 to -0.50)	-0.37 (-0.56 to -0.18)	-0.22 (-0.47 to 0.04)	-0.02 (-0.23 to 0.19)	**Basal insulin**	
-1.96 (-2.11 to -1.82)	-1.84 (-1.99 to -1.69)	-1.60 (-1.75 to -1.44)	-1.59 (-1.95 to -1.22)	-1.39 (-1.52 to -1.26)	-1.09 (-1.24 to -0.94)	-0.93 (-1.17 to -0.69)	-0.73 (-0.91 to -0.56)	-0.72 (-0.90 to -0.53)	**Placebo**
Treatment estimates are mean differences (MDs) and 95% CIs in the column-defining treatment compared to the row-defining treatment. Values lower than 0 favour the column-defining treatment.									

ESM Table 8 Confidence in effect estimates for the change from baseline in HbA1c (mmol/mol) in comparisons between tirzepatide, semaglutide, and placebo

**Comparison**	**Within-study bias**	**Reporting bias**	**Indirectness**	**Imprecision**	**Heterogeneity**	**Incoherence**	**Confidence rating**
Placebo:Semaglutide 0.5 mg	No concerns	Low risk	No concerns	No concerns	No concerns	No concerns	High
Placebo:Semaglutide 1.0 mg	No concerns	Low risk	No concerns	No concerns	No concerns	No concerns	High
Placebo:Tirzepatide 10 mg	No concerns	Low risk	No concerns	No concerns	No concerns	No concerns	High
Placebo:Tirzepatide 15 mg	No concerns	Low risk	No concerns	No concerns	No concerns	No concerns	High
Placebo:Tirzepatide 5 mg	No concerns	Low risk	No concerns	No concerns	No concerns	No concerns	High
Semaglutide 0.5 mg:Semaglutide 1.0 mg	No concerns	Low risk	No concerns	No concerns	Some concerns	No concerns	Moderate
Semaglutide 1.0 mg:Semaglutide 2.0 mg	No concerns	Low risk	Some concerns	Some concerns	No concerns	No concerns	Low
Semaglutide 1.0 mg:Tirzepatide 10 mg	No concerns	Low risk	No concerns	No concerns	No concerns	No concerns	High
Semaglutide 1.0 mg:Tirzepatide 15 mg	No concerns	Low risk	No concerns	No concerns	No concerns	No concerns	High
Semaglutide 1.0 mg:Tirzepatide 5 mg	No concerns	Low risk	No concerns	No concerns	Some concerns	No concerns	Moderate
Tirzepatide 10 mg:Tirzepatide 15 mg	No concerns	Low risk	No concerns	No concerns	Some concerns	No concerns	Moderate
Tirzepatide 10 mg:Tirzepatide 5 mg	No concerns	Low risk	No concerns	No concerns	Some concerns	No concerns	Moderate
Tirzepatide 15 mg:Tirzepatide 5 mg	No concerns	Low risk	No concerns	No concerns	Some concerns	No concerns	Moderate
Placebo:Semaglutide 2.0 mg	No concerns	Low risk	Some concerns	No concerns	No concerns	No concerns	Moderate
Semaglutide 0.5 mg:Semaglutide 2.0 mg	No concerns	Low risk	Some concerns	No concerns	Some concerns	No concerns	Low
Semaglutide 0.5 mg:Tirzepatide 10 mg	No concerns	Low risk	No concerns	No concerns	No concerns	No concerns	High
Semaglutide 0.5 mg:Tirzepatide 15 mg	No concerns	Low risk	No concerns	No concerns	No concerns	No concerns	High
Semaglutide 0.5 mg:Tirzepatide 5 mg	No concerns	Low risk	No concerns	No concerns	No concerns	No concerns	High
Semaglutide 2.0 mg:Tirzepatide 10 mg	No concerns	Low risk	Some concerns	Some concerns	No concerns	No concerns	Low
Semaglutide 2.0 mg:Tirzepatide 15 mg	No concerns	Low risk	Some concerns	Some concerns	No concerns	No concerns	Low
Semaglutide 2.0 mg:Tirzepatide 5 mg	No concerns	Low risk	Some concerns	No concerns	Major concerns	No concerns	Very low

ESM Table 9 Network meta-analysis results for achievement of an HbA1c target of ≤48 mmol/mol (≤6.5%)

**Tirzepatide 15 mg**									
1.03 (0.96 to 1.09)	**Tirzepatide 10 mg**								
1.15 (1.07 to 1.23)	1.12 (1.04 to 1.20)	**Tirzepatide 5 mg**							
0.91 (0.69 to 1.18)	0.88 (0.68 to 1.15)	0.79 (0.61 to 1.03)	**Semaglutide 2.0 mg**						
1.22 (1.08 to 1.38)	1.19 (1.05 to 1.34)	1.06 (0.94 to 1.20)	1.34 (1.06 to 1.70)	**Semaglutide 1.0 mg**					
1.55 (1.35 to 1.80)	1.52 (1.31 to 1.75)	1.35 (1.17 to 1.57)	1.71 (1.33 to 2.21)	1.28 (1.17 to 1.40)	**Semaglutide 0.5 mg**				
2.30 (1.98 to 2.66)	2.24 (1.93 to 2.59)	2.00 (1.73 to 2.32)	2.53 (1.94 to 3.31)	1.88 (1.66 to 2.13)	1.48 (1.28 to 1.70)	**GLP-1 RA**			
2.58 (2.28 to 2.93)	2.52 (2.22 to 2.85)	2.25 (1.98 to 2.55)	2.85 (2.15 to 3.77)	2.12 (1.82 to 2.47)	1.66 (1.40 to 1.97)	1.13 (0.94 to 1.34)	**Basal insulin**		
2.40 (2.02 to 2.86)	2.34 (1.97 to 2.79)	2.09 (1.76 to 2.50)	2.65 (1.97 to 3.56)	1.97 (1.65 to 2.35)	1.55 (1.27 to 1.88)	1.05 (0.85 to 1.28)	0.93 (0.76 to 1.14)	**Prandial insulin**	
7.01 (5.73 to 8.57)	6.83 (5.58 to 8.36)	6.11 (4.97 to 7.50)	7.73 (5.62 to 10.63)	5.75 (4.65 to 7.12)	4.51 (3.60 to 5.64)	3.05 (2.42 to 3.85)	2.71 (2.16 to 3.41)	2.92 (2.26 to 3.77)	**Placebo**
Treatment estimates are risk ratios (RRs) and 95% CIs in the column-defining treatment compared to the row-defining treatment. RRs greater than 1 favour the column-defining treatment.									

ESM Table 10 Network meta-analysis results for achievement of an HbA1c target of <53 mmol/mol (<7.0%)

**Tirzepatide 15 mg**									
1.00 (0.96 to 1.05)	**Tirzepatide 10 mg**								
1.09 (1.03 to 1.14)	1.08 (1.03 to 1.14)	**Tirzepatide 5 mg**							
0.92 (0.77 to 1.11)	0.92 (0.76 to 1.11)	0.85 (0.70 to 1.02)	**Semaglutide 2.0 mg**						
1.08 (1.00 to 1.18)	1.08 (0.99 to 1.17)	1.00 (0.91 to 1.08)	1.17 (0.99 to 1.38)	**Semaglutide 1.0 mg**					
1.25 (1.13 to 1.38)	1.25 (1.13 to 1.38)	1.15 (1.04 to 1.27)	1.35 (1.13 to 1.62)	1.15 (1.08 to 1.23)	**Semaglutide 0.5 mg**				
1.59 (1.44 to 1.76)	1.59 (1.44 to 1.75)	1.47 (1.33 to 1.62)	1.72 (1.43 to 2.08)	1.47 (1.35 to 1.60)	1.27 (1.15 to 1.41)	**GLP-1 RA**			
1.82 (1.67 to 1.98)	1.82 (1.67 to 1.98)	1.68 (1.54 to 1.83)	1.97 (1.62 to 2.40)	1.68 (1.52 to 1.86)	1.46 (1.30 to 1.63)	1.14 (1.01 to 1.29)	**Basal insulin**		
1.73 (1.53 to 1.96)	1.73 (1.53 to 1.96)	1.60 (1.41 to 1.81)	1.88 (1.53 to 2.31)	1.60 (1.42 to 1.81)	1.39 (1.21 to 1.59)	1.09 (0.95 to 1.25)	0.95 (0.83 to 1.10)	**Prandial insulin**	
3.70 (3.26 to 4.20)	3.69 (3.25 to 4.19)	3.41 (2.99 to 3.88)	4.01 (3.24 to 4.95)	3.42 (3.00 to 3.90)	2.96 (2.57 to 3.41)	2.32 (2.01 to 2.69)	2.03 (1.76 to 2.35)	2.13 (1.81 to 2.52)	**Placebo**
Treatment estimates are risk ratios (RRs) and 95% CIs in the column-defining treatment compared to the row-defining treatment. RRs greater than 1 favour the column-defining treatment.									

ESM Table 11 Network meta-analysis results for the change from baseline in body weight (kg)

**Tirzepatide 15 mg**									
-1.87 (-2.85 to -0.90)	**Tirzepatide 10 mg**								
-4.30 (-5.31 to -3.29)	-2.43 (-3.44 to -1.41)	**Tirzepatide 5 mg**							
-4.60 (-7.94 to -1.26)	-2.73 (-6.08 to 0.63)	-0.30 (-3.67 to 3.07)	**Semaglutide 2.0 mg**						
-5.40 (-6.64 to -4.15)	-3.53 (-4.80 to -2.25)	-1.10 (-2.41 to 0.21)	-0.80 (-3.90 to 2.30)	**Semaglutide 1.0 mg**					
-7.05 (-8.47 to -5.63)	-5.18 (-6.63 to -3.74)	-2.75 (-4.23 to -1.28)	-2.46 (-5.72 to 0.81)	-1.66 (-2.66 to -0.65)	**Semaglutide 0.5 mg**				
-9.04 (-10.51 to -7.58)	-7.17 (-8.65 to -5.69)	-4.74 (-6.25 to -3.24)	-4.44 (-7.79 to -1.10)	-3.64 (-4.89 to -2.39)	-1.99 (-3.31 to -0.66)	**GLP-1 RA**			
-9.57 (-10.78 to -8.36)	-7.70 (-8.94 to -6.46)	-5.27 (-6.56 to -3.98)	-4.97 (-8.26 to -1.68)	-4.17 (-5.26 to -3.09)	-2.52 (-3.78 to -1.26)	-0.53 (-1.97 to 0.92)	**Placebo**		
-12.43 (-13.86 to -11.01)	-10.56 (-12.00 to -9.13)	-8.14 (-9.58 to -6.69)	-7.84 (-11.32 to -4.36)	-7.04 (-8.61 to -5.46)	-5.38 (-7.07 to -3.70)	-3.39 (-5.18 to -1.61)	-2.86 (-4.49 to -1.24)	**Basal insulin**	
-13.42 (-15.51 to -11.33)	-11.55 (-13.64 to -9.45)	-9.12 (-11.23 to -7.01)	-8.82 (-12.55 to -5.09)	-8.02 (-10.09 to -5.95)	-6.37 (-8.60 to -4.14)	-4.38 (-6.67 to -2.09)	-3.85 (-6.02 to -1.68)	-0.99 (-3.35 to 1.38)	**Prandial insulin**
Treatment estimates are mean differences (MDs) and 95% CIs in the column-defining treatment compared to the row-defining treatment. Values lower than 0 favour the column-defining treatment.									

ESM Table 12 Confidence in effect estimates for the change from baseline in in body weight (kg) in comparisons between tirzepatide to semaglutide to and placebo

Comparison	Within-study bias	Reporting bias	Indirectness	Imprecision	Heterogeneity	Incoherence	Confidence rating
Placebo:Semaglutide 0.5 mg	No concerns	Low risk	No concerns	No concerns	Some concerns	No concerns	Moderate
Placebo:Semaglutide 1.0 mg	No concerns	Low risk	No concerns	No concerns	No concerns	No concerns	High
Placebo:Tirzepatide 10 mg	No concerns	Low risk	No concerns	No concerns	No concerns	No concerns	High
Placebo:Tirzepatide 15 mg	No concerns	Low risk	No concerns	No concerns	No concerns	No concerns	High
Placebo:Tirzepatide 5 mg	No concerns	Low risk	No concerns	No concerns	No concerns	No concerns	High
Semaglutide 0.5 mg:Semaglutide 1.0 mg	No concerns	Low risk	No concerns	No concerns	Some concerns	No concerns	Moderate
Semaglutide 1.0 mg:Semaglutide 2.0 mg	No concerns	Low risk	Some concerns	No concerns	Some concerns	No concerns	Low
Semaglutide 1.0 mg:Tirzepatide 10 mg	Some concerns	Low risk	No concerns	No concerns	No concerns	No concerns	Moderate
Semaglutide 1.0 mg:Tirzepatide 15 mg	No concerns	Low risk	No concerns	No concerns	No concerns	No concerns	High
Semaglutide 1.0 mg:Tirzepatide 5 mg	Some concerns	Low risk	No concerns	No concerns	No concerns	No concerns	Moderate
Tirzepatide 10 mg:Tirzepatide 15 mg	Some concerns	Low risk	No concerns	No concerns	Some concerns	No concerns	Low
Tirzepatide 10 mg:Tirzepatide 5 mg	Some concerns	Low risk	No concerns	No concerns	Some concerns	No concerns	Low
Tirzepatide 15 mg:Tirzepatide 5 mg	Some concerns	Low risk	No concerns	No concerns	No concerns	Major concerns	Low
Placebo:Semaglutide 2.0 mg	No concerns	Low risk	Some concerns	No concerns	No concerns	No concerns	Moderate
Semaglutide 0.5 mg:Semaglutide 2.0 mg	No concerns	Low risk	Some concerns	Some concerns	No concerns	No concerns	Low
Semaglutide 0.5 mg:Tirzepatide 10 mg	No concerns	Low risk	No concerns	No concerns	No concerns	No concerns	High
Semaglutide 0.5 mg:Tirzepatide 15 mg	No concerns	Low risk	No concerns	No concerns	No concerns	No concerns	High
Semaglutide 0.5 mg:Tirzepatide 5 mg	Some concerns	Low risk	No concerns	No concerns	Some concerns	No concerns	Low
Semaglutide 2.0 mg:Tirzepatide 10 mg	No concerns	Low risk	Some concerns	Some concerns	No concerns	No concerns	Low
Semaglutide 2.0 mg:Tirzepatide 15 mg	No concerns	Low risk	Some concerns	No concerns	Some concerns	No concerns	Low
Semaglutide 2.0 mg:Tirzepatide 5 mg	No concerns	Low risk	Some concerns	No concerns	Some concerns	No concerns	Low

ESM Table 13 Network meta-analysis results for achievement of at least a 10% body weight reduction

**Tirzepatide 15 mg**									
1.19 (1.09 to 1.30)	**Tirzepatide 10 mg**								
1.85 (1.67 to 2.05)	1.56 (1.40 to 1.73)	**Tirzepatide 5 mg**							
1.76 (1.21 to 2.55)	1.48 (1.01 to 2.15)	0.95 (0.65 to 1.38)	**Semaglutide 2.0 mg**						
2.20 (1.77 to 2.72)	1.85 (1.49 to 2.29)	1.19 (0.95 to 1.48)	1.25 (0.92 to 1.70)	**Semaglutide 1.0 mg**					
4.39 (3.32 to 5.80)	3.69 (2.79 to 4.88)	2.37 (1.78 to 3.15)	2.50 (1.74 to 3.59)	2.00 (1.65 to 2.42)	**Semaglutide 0.5 mg**				
10.32 (7.51 to 14.19)	8.68 (6.31 to 11.94)	5.58 (4.04 to 7.70)	5.88 (3.91 to 8.84)	4.70 (3.59 to 6.15)	2.35 (1.72 to 3.21)	**GLP-1 RA**			
32.03 (22.18 to 46.27)	26.94 (18.63 to 38.93)	17.30 (11.93 to 25.08)	18.25 (11.01 to 30.23)	14.59 (9.77 to 21.79)	7.30 (4.73 to 11.28)	3.10 (1.94 to 4.95)	**Basal insulin**		
15.01 (10.47 to 21.53)	12.62 (8.80 to 18.12)	8.11 (5.63 to 11.67)	8.55 (5.16 to 14.17)	6.84 (4.58 to 10.21)	3.42 (2.20 to 5.31)	1.45 (0.91 to 2.32)	0.47 (0.28 to 0.78)	**Prandial insulin**	
10.51 (7.55 to 14.64)	8.84 (6.35 to 12.32)	5.68 (4.04 to 7.98)	5.99 (3.73 to 9.61)	4.79 (3.34 to 6.87)	2.40 (1.61 to 3.57)	1.02 (0.66 to 1.57)	0.33 (0.20 to 0.53)	0.70 (0.43 to 1.13)	**Placebo**
Treatment estimates are risk ratios (RRs) and 95% CIs in the column-defining treatment compared to the row-defining treatment. RRs greater than 1 favour the column-defining treatment									

ESM Table 14 Network meta-analysis results for the incidence of nausea

**Tirzepatide 15 mg**									
1.17 (1.02 to 1.34)	**Tirzepatide 10 mg**								
1.69 (1.45 to 1.98)	1.45 (1.23 to 1.70)	**Tirzepatide 5 mg**							
1.25 (0.79 to 1.97)	1.07 (0.68 to 1.69)	0.74 (0.47 to 1.17)	**Semaglutide 2.0 mg**						
1.24 (1.00 to 1.53)	1.06 (0.85 to 1.31)	0.73 (0.58 to 0.92)	0.99 (0.66 to 1.48)	**Semaglutide 1.0 mg**					
1.43 (1.12 to 1.83)	1.22 (0.95 to 1.57)	0.85 (0.65 to 1.10)	1.14 (0.74 to 1.76)	1.16 (0.99 to 1.35)	**Semaglutide 0.5 mg**				
1.86 (1.44 to 2.40)	1.58 (1.22 to 2.06)	1.10 (0.84 to 1.44)	1.48 (0.95 to 2.32)	1.50 (.23 to 1.83)	1.30 (1.03 to 1.63)	**GLP-1 RA**			
9.93 (7.13 to 13.81)	8.47 (6.07 to 11.83)	5.86 (4.17 to 8.24)	7.93 (4.65 to 13.52)	8.03 (5.65 to 11.40)	6.93 (4.81 to 10.00)	5.35 (3.64 to 7.85)	**Basal insulin**		
22.19 (12.77 to 38.59)	18.95 (10.88 to 33.01)	13.11 (7.49 to 22.93)	17.73 (8.96 to 35.10)	17.95 (10.34 to 31.17)	15.50 (8.78 to 27.39)	11.96 (6.71 to 21.31)	2.24 (1.19 to 4.19)	**Prandial insulin**	
3.51 (2.72 to 4.51)	2.99 (2.31 to 3.87)	2.07 (1.58 to 2.71)	2.80 (1.77 to 4.42)	2.84 (2.29 to 3.52)	2.45 (1.94 to 3.09)	1.89 (1.43 to 2.49)	0.35 (0.24 to 0.52)	0.16 (0.09 to 0.28)	**Placebo**

Treatment estimates are risk ratios (RRs) and 95% CIs in the column-defining treatment compared to the row-defining treatment. RRs lower than 1 favour the column-defining treatment

ESM Table 15 Network meta-analysis results for the incidence of vomiting

**Tirzepatide 15 mg**									
1.20 (1.02 to 1.42)	**Tirzepatide 10 mg**								
1.82 (1.49 to 2.23)	1.52 (1.23 to 1.87)	**Tirzepatide 5 mg**							
1.20 (0.71 to 2.04)	1.00 (0.59 to 1.70)	0.66 (0.38 to 1.14)	**Semaglutide 2.0 mg**						
1.39 (1.07 to 1.82)	1.16 (0.88 to 1.52)	0.77 (0.57 to 1.03)	1.16 (0.73 to 1.83)	**Semaglutide 1.0 mg**					
1.87 (1.39 to 2.53)	1.56 (1.15 to 2.12)	1.03 (0.74 to 1.43)	1.56 (0.96 to 2.53)	1.34 (1.14 to 1.59)	**Semaglutide 0.5 mg**				
2.17 (1.53 to 3.06)	1.80 (1.27 to 2.56)	1.19 (0.82 to 1.72)	1.80 (1.07 to 3.03)	1.55 (1.21 to 2.00)	1.16 (0.87 to 1.53)	**GLP-1 RA**			
6.02 (4.13 to 8.78)	5.01 (3.43 to 7.33)	3.30 (2.22 to 4.92)	5.01 (2.73 to 9.17)	4.32 (2.90 to 6.43)	3.21 (2.12 to 4.88)	2.78 (1.76 to 4.40)	**Basal insulin**		
16.30 (8.14 to 32.65)	13.57 (6.75 to 27.28)	8.95 (4.41 to 18.16)	13.55 (5.95 to 30.89)	11.70 (5.89 to 23.23)	8.70 (4.30 to 17.59)	7.53 (3.64 to 15.56)	2.71 (1.26 to 5.83)	**Prandial insulin**	
4.36 (3.20 to 5.93)	3.63 (2.65 to 4.97)	2.39 (1.71 to 3.35)	3.62 (2.18 to 6.01)	3.13 (2.50 to 3.90)	2.33 (1.82 to 2.97)	2.01 (1.45 to 2.79)	0.72 (0.47 to 1.12)	0.27 (0.13 to 0.55)	**Placebo**
Treatment estimates are risk ratios (RRs) and 95% CIs in the column-defining treatment compared to the row-defining treatment. RRs lower than 1 favour the column-defining treatment									

ESM Table 16 Network meta-analysis results for the incidence of diarrhoea

**Tirzepatide 15 mg**									
0.99 (0.86 to 1.13)	**Tirzepatide 10 mg**								
1.19 (1.03 to 1.38)	1.21 (1.04 to 1.40)	**Tirzepatide 5 mg**							
1.20 (0.73 to 1.98)	1.22 (0.73 to 2.01)	1.01 (0.61 to 1.67)	**Semaglutide 2.0 mg**						
1.29 (1.04 to 1.59)	1.31 (1.05 to 1.62)	1.08 (0.87 to 1.35)	1.07 (0.68 to 1.69)	**Semaglutide 1.0 mg**					
1.30 (1.02 to 1.65)	1.31 (1.03 to 1.68)	1.09 (0.84 to 1.40)	1.08 (0.67 to 1.75)	1.01 (0.86 to 1.17)	**Semaglutide 0.5 mg**				
1.63 (1.24 to 2.14)	1.65 (1.26 to 2.18)	1.37 (1.04 to 1.81)	1.36 (0.82 to 2.25)	1.27 (1.02 to 1.57)	1.26 (0.99 to 1.61)	**GLP-1 RA**			
5.00 (3.81 to 6.56)	5.06 (3.85 to 6.66)	4.20 (3.17 to 5.55)	4.16 (2.41 to 7.19)	3.88 (2.87 to 5.25)	3.86 (2.80 to 5.31)	3.06 (2.15 to 4.35)	**Basal insulin**		
4.48 (3.02 to 6.63)	4.53 (3.06 to 6.72)	3.76 (2.53 to 5.59)	3.73 (2.06 to 6.76)	3.47 (2.37 to 5.09)	3.45 (2.30 to 5.19)	2.74 (1.78 to 4.22)	0.90 (0.57 to 1.41)	**Prandial insulin**	
2.16 (1.71 to 2.72)	2.18 (1.73 to 2.76)	1.81 (1.42 to 2.32)	1.80 (1.09 to 2.95)	1.67 (1.37 to 2.04)	1.66 (1.34 to 2.07)	1.32 (1.00 to 1.74)	0.43 (0.31 to 0.60)	0.48 (0.32 to 0.73)	**Placebo**
Treatment estimates are risk ratios (RRs) and 95% CIs in the column-defining treatment compared to the row-defining treatment. RRs lower than 1 favour the column-defining treatment									

ESM Table 17 Network meta-analysis results for treatment discontinuation due to gastrointestinal adverse events

**Tirzepatide 15 mg**									
1.21 (0.92 to 1.58)	**Tirzepatide 10 mg**								
1.67 (1.23 to 2.27)	1.38 (1.00 to 1.90)	**Tirzepatide 5 mg**							
1.20 (0.48 to 2.96)	0.99 (0.40 to 2.46)	0.72 (0.29 to 1.80)	**Semaglutide 2.0 mg**						
1.47 (0.85 to 2.55)	1.22 (0.70 to 2.13)	0.89 (0.50 to 1.57)	1.23 (0.60 to 2.54)	**Semaglutide 1.0 mg**					
2.14 (1.19 to 3.83)	1.77 (0.98 to 3.21)	1.28 (0.70 to 2.36)	1.79 (0.84 to 3.80)	1.45 (1.15 to 1.82)	**Semaglutide 0.5 mg**				
3.14 (1.65 to .96)	2.60 (1.36 to 4.98)	1.88 (0.97 to 3.65)	2.63 (1.17 to 5.88)	2.13 (1.48 to 3.06)	1.47 (0.99 to 2.19)	**GLP-1 RA**			
36.66 (10.39 to 129.38)	30.36 (8.58 to 107.34)	22.00 (6.17 to 78.49)	30.66 (6.82 to 137.83)	24.86 (6.65 to 92.96)	17.17 (4.53 to 65.10)	11.68 (2.99 to 45.56)	**Basal insulin**		
45.72 (2.60 to 804.23)	37.86 (2.15 to 667.29)	27.44 (1.55 to 485.10)	38.23 (2.09 to 698.65)	31.00 (1.86 to 517.28)	21.41 (1.27 to 360.54)	14.57 (0.85 to 248.74)	1.25 (0.06 to 27.92)	**Prandial insulin**	
10.65 (5.58 to 20.35)	8.82 (4.56 to 17.04)	6.39 (3.26 to 12.52)	8.91 (3.82 to 20.74)	7.22 (4.64 to 11.23)	4.99 (3.14 to 7.92)	3.39 (1.93 to 5.96)	0.29 (0.07 to 1.14)	0.23 (0.01 to 4.02)	**Placebo**
Treatment estimates are risk ratios (RRs) and 95% CIs in the column-defining treatment compared to the row-defining treatment. RRs lower than 1 favour the column-defining treatment									

ESM Table 18 Network meta-analysis results for the incidence of serious adverse events

**Tirzepatide 15 mg**									
1.04 (0.84 to 1.29)	**Tirzepatide 10 mg**								
1.00 (0.79 to 1.26)	0.96 (0.76 to 1.21)	**Tirzepatide 5 mg**							
1.20 (0.60 to 2.39)	1.15 (0.58 to 2.29)	1.20 (0.60 to 2.40)	**Semaglutide 2.0 mg**						
1.01 (0.75 to 1.35)	0.97 (0.72 to 1.29)	1.01 (0.75 to 1.36)	0.84 (0.45 to 1.57)	**Semaglutide 1.0 mg**					
0.99 (0.72 to 1.36)	0.95 (0.69 to 1.31)	0.99 (0.71 to 1.38)	0.83 (0.43 to 1.60)	0.98 (0.81 to 1.20)	**Semaglutide 0.5 mg**				
0.93 (0.64 to 1.34)	0.89 (0.62 to 1.28)	0.93 (0.64 to 1.35)	0.77 (0.39 to 1.54)	0.92 (0.69 to 1.23)	0.93 (0.68 to 1.29)	**GLP-1 RA**			
0.88 (0.69 to 1.13)	0.85 (0.66 to 1.09)	0.88 (0.68 to 1.14)	0.74 (0.37 to 1.49)	0.88 (0.64 to 1.20)	0.89 (0.63 to 1.25)	0.95 (0.64 to 1.41)	**Basal insulin**		
0.67 (0.47 to 0.94)	0.64 (0.46 to 0.91)	0.67 (0.47 to 0.95)	0.56 (0.28 to 1.13)	0.66 (0.48 to 0.91)	0.68 (0.47 to 0.97)	0.72 (0.48 to 1.09)	0.76 (0.52 to 1.11)	**Prandial insulin**	
0.95 (0.70 to 1.28)	0.91 (0.67 to 1.23)	0.95 (0.69 to 1.30)	0.79 (0.41 to 1.54)	0.94 (0.76 to 1.17)	0.96 (0.76 to 1.21)	1.02 (0.73 to 1.44)	1.08 (0.77 to 1.50)	1.42 (0.99 to 2.03)	**Placebo**
Treatment estimates are risk ratios (RRs) and 95% CIs in the column-defining treatment compared to the row-defining treatment. RRs lower than 1 favour the column-defining treatment									

ESM Figures

ESM Fig. 1 PRISMA flow diagram of the study selection process

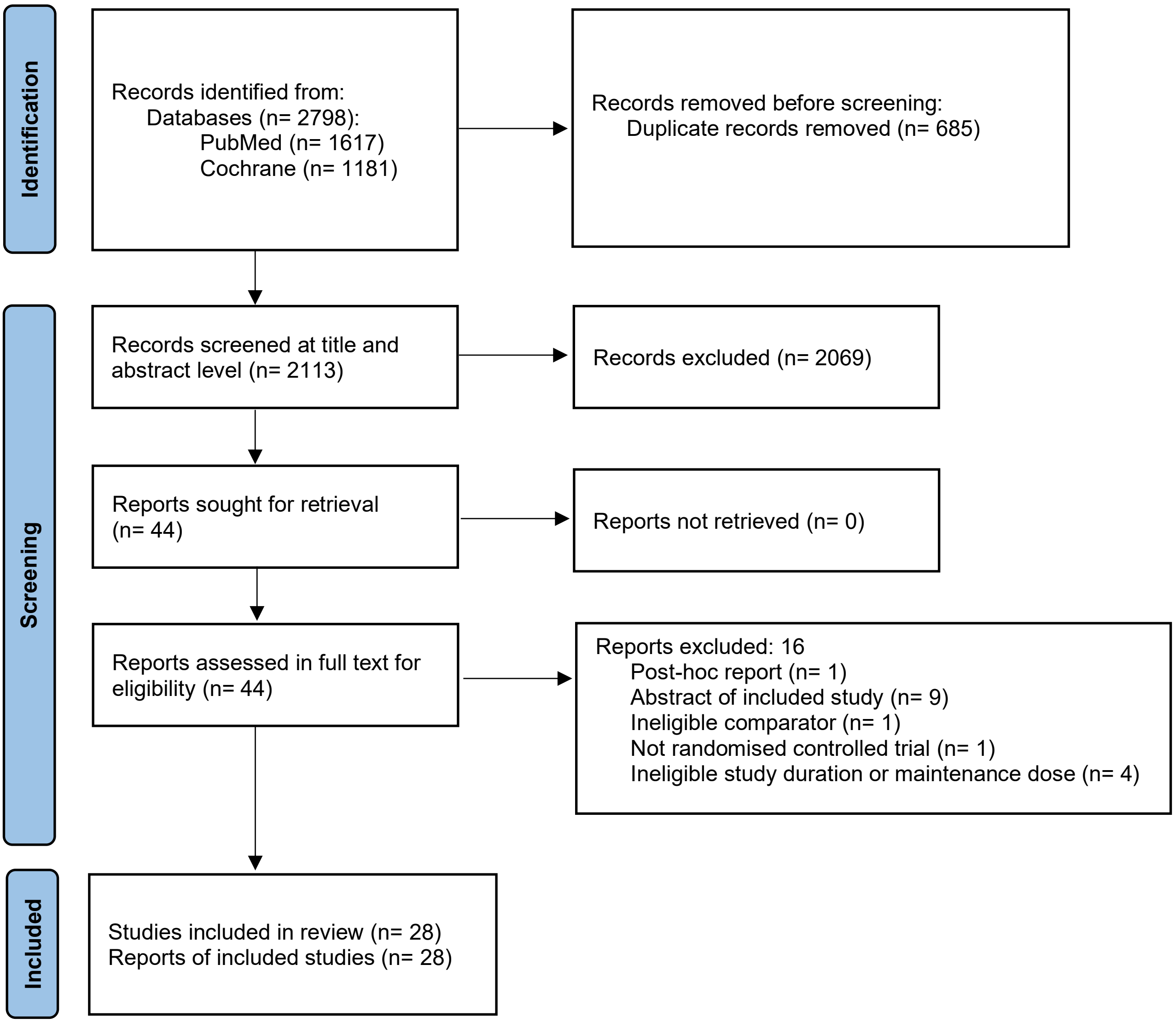

ESM Fig. 2 Assessment of transitivity based on the distribution of mean baseline HbA1c (%)

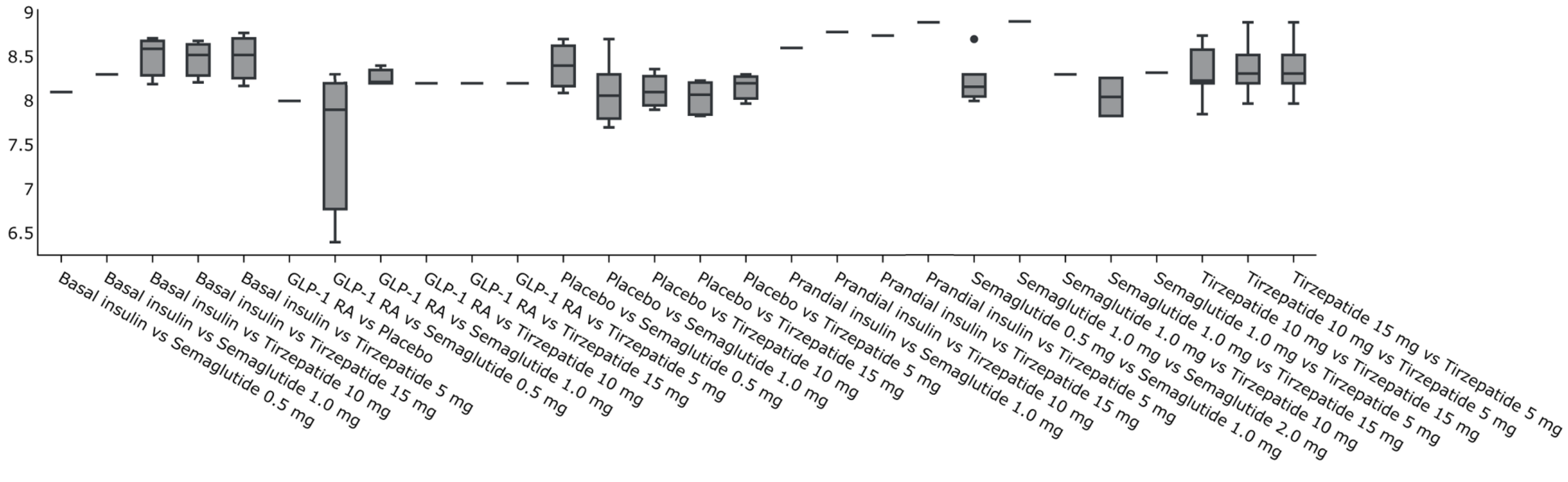

ESM Fig. 3 Assessment of transitivity based on the distribution of mean baseline body weight (kg)

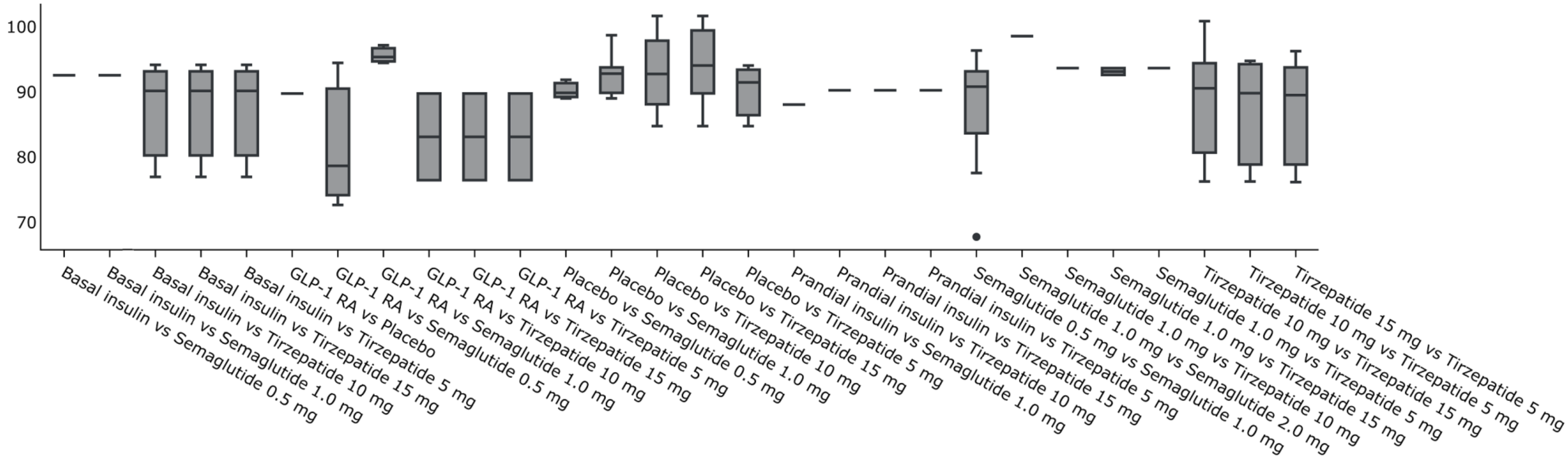

ESM Fig. 4 Comparison-adjusted funnel plot for the change from baseline in HbA1c (mmol/mol)

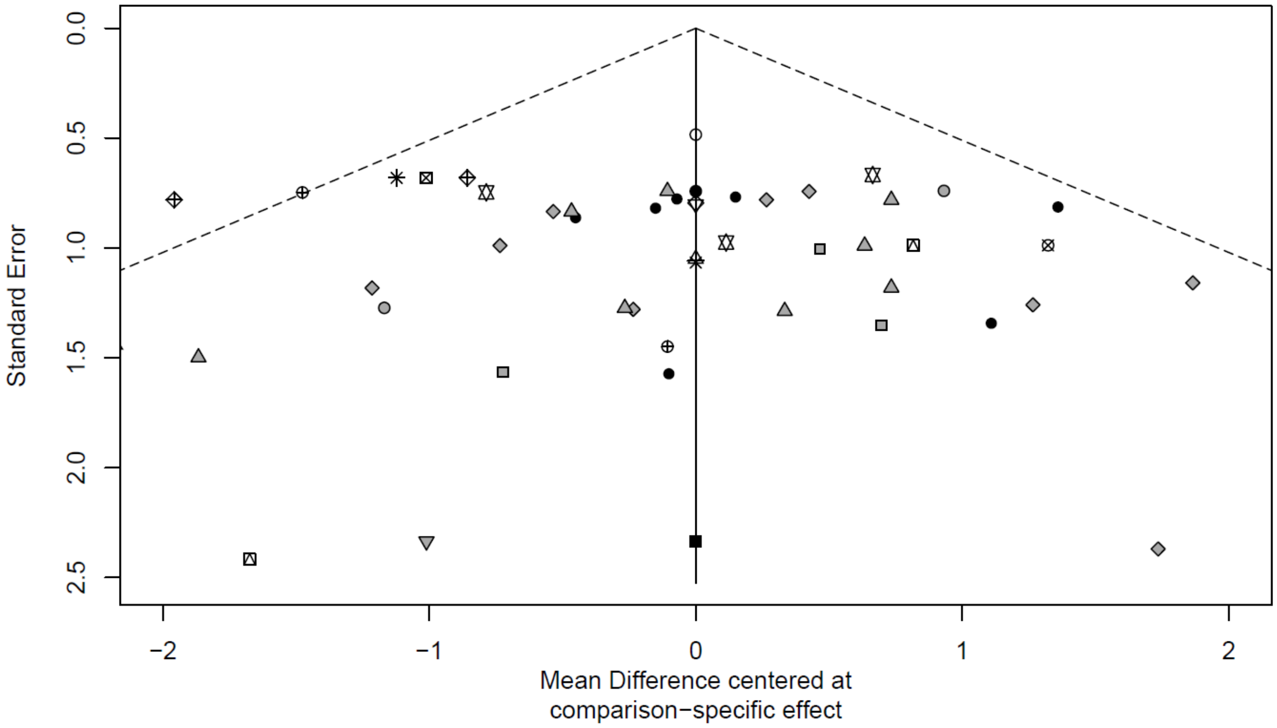

ESM Fig. 5 Comparison-adjusted funnel plot for the change from baseline in body weight (kg)

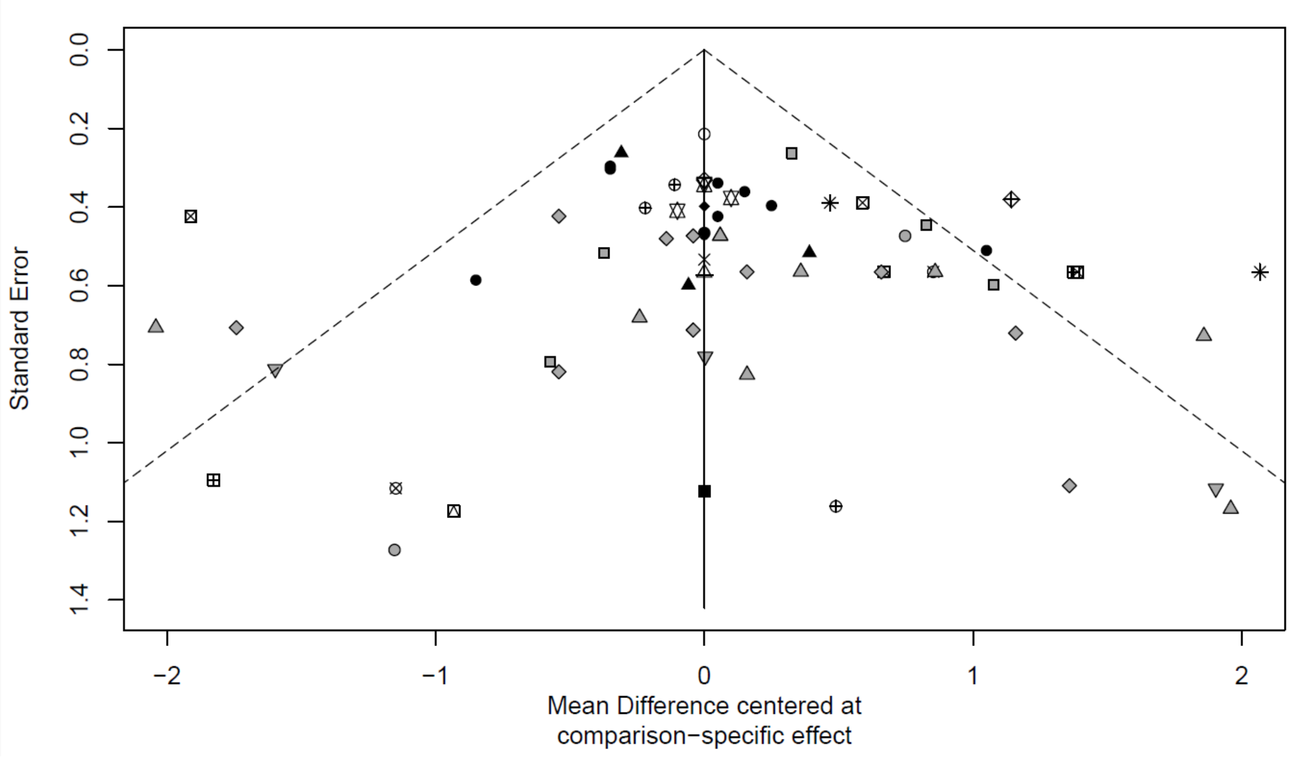

ESM Fig. 6 Network meta-analysis results compared to placebo for the change from baseline in HbA1c (%). MD, mean difference

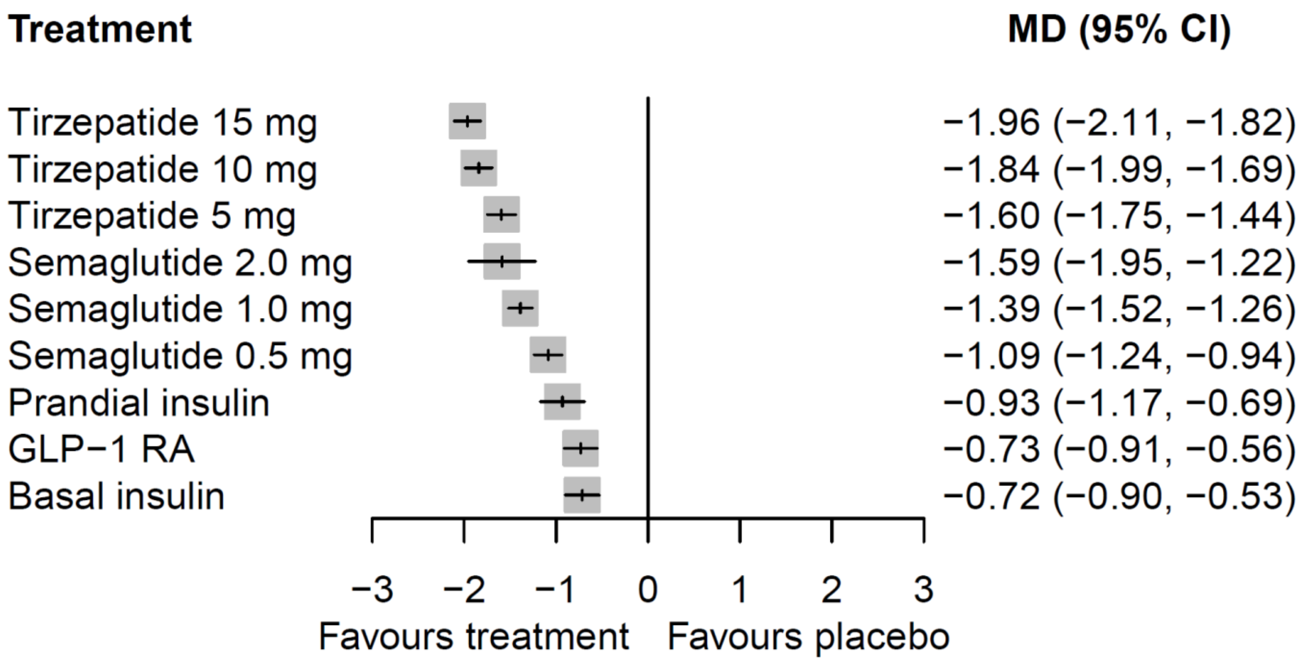

ESM Fig. 7 Treatment ranking (P-scores) for the change from baseline in HbA1c (mmol/mol). P-scores range from 0 to 1 to and can be interpreted as an average degree of probability for a treatment to be better than the other treatments in the network

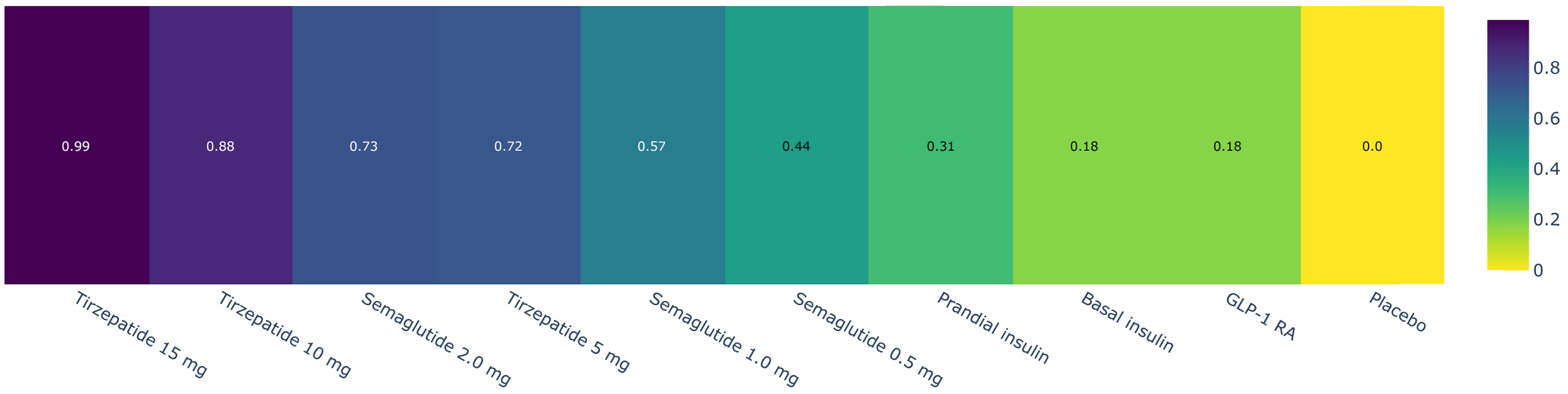

ESM Fig. 8 Treatment ranking (P-scores) for the change from baseline in body weight (kg). P-scores range from 0 to 1 to and can be interpreted as an average degree of probability for a treatment to be better than the other treatments in the network

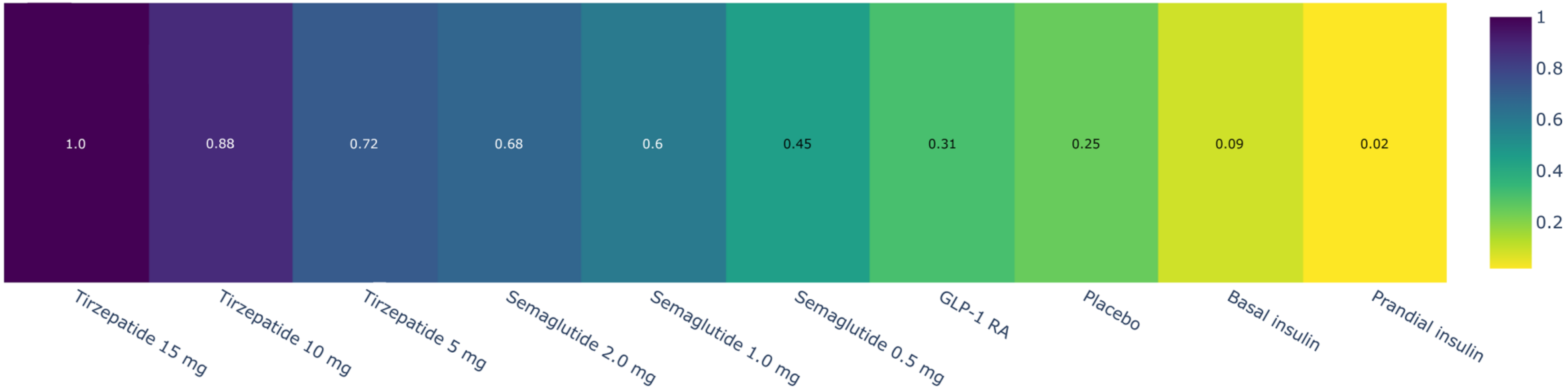

ESM Fig. 9 Network meta-analysis results compared to placebo for the incidence of nausea. RR, risk ratio

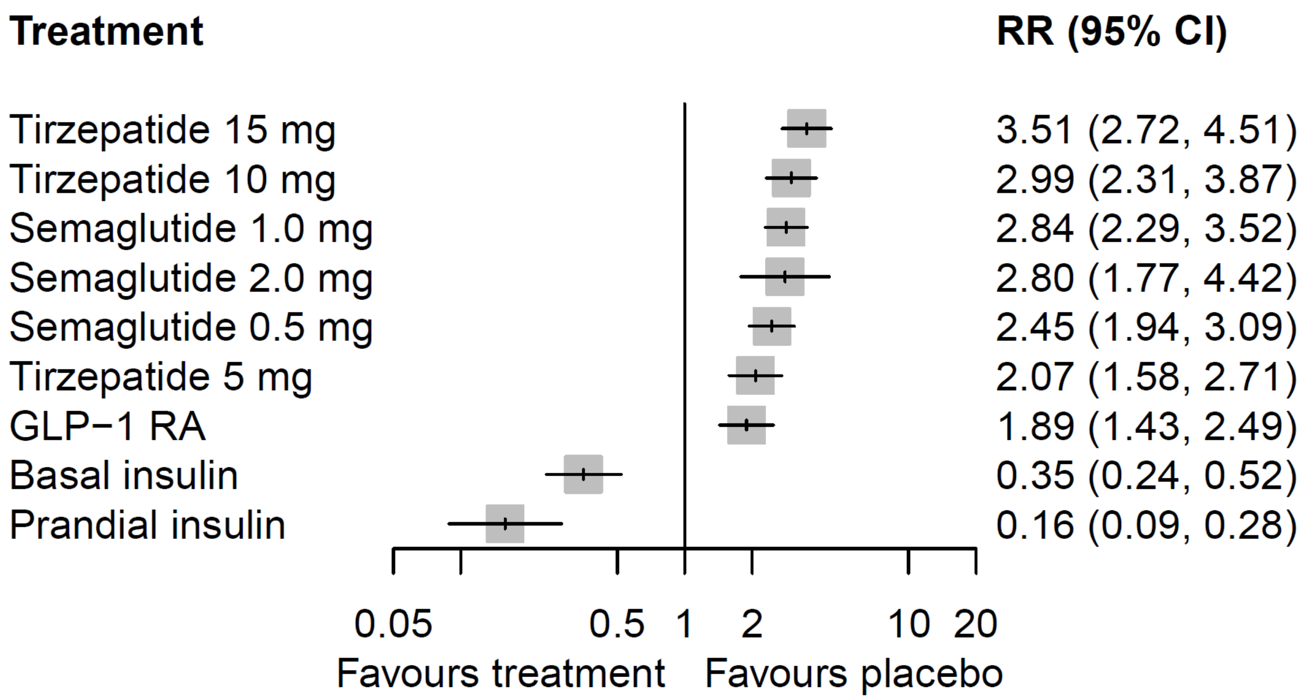

ESM Fig. 10 Network meta-analysis results compared to placebo for the incidence of vomiting. RR, risk ratio

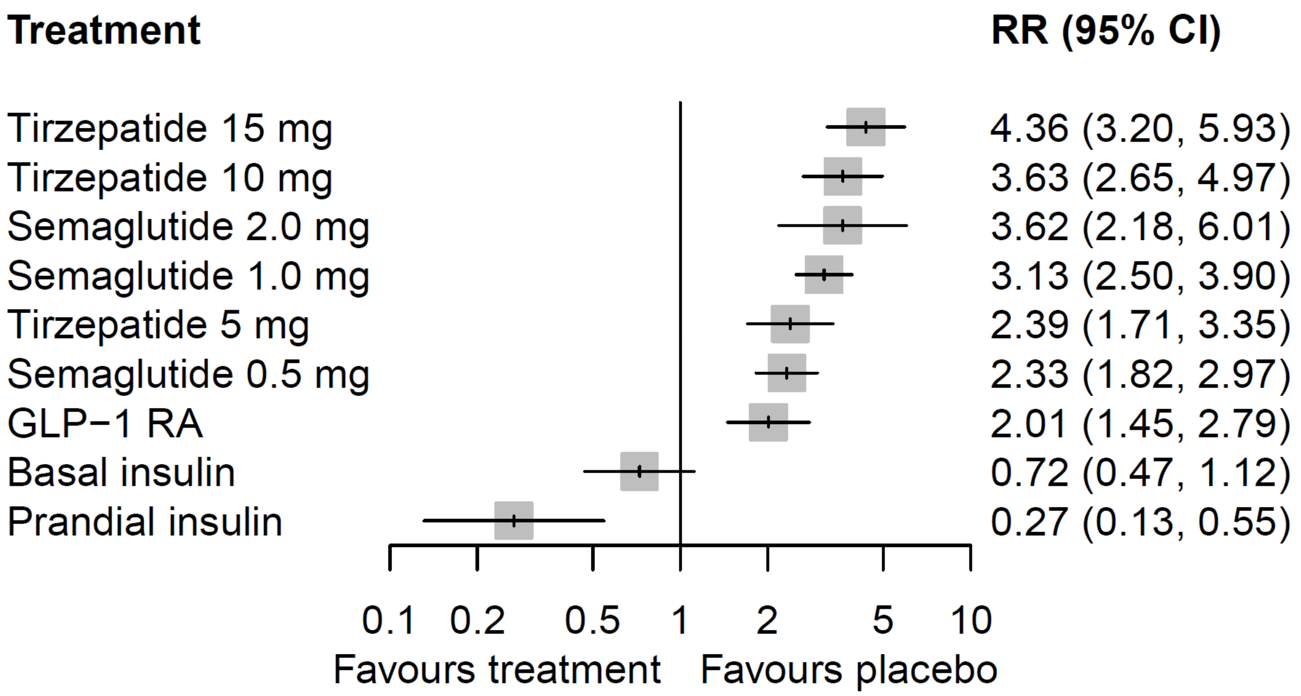

ESM Fig. 11 Network meta-analysis results compared to placebo for the incidence of diarrhoea. RR, risk ratio

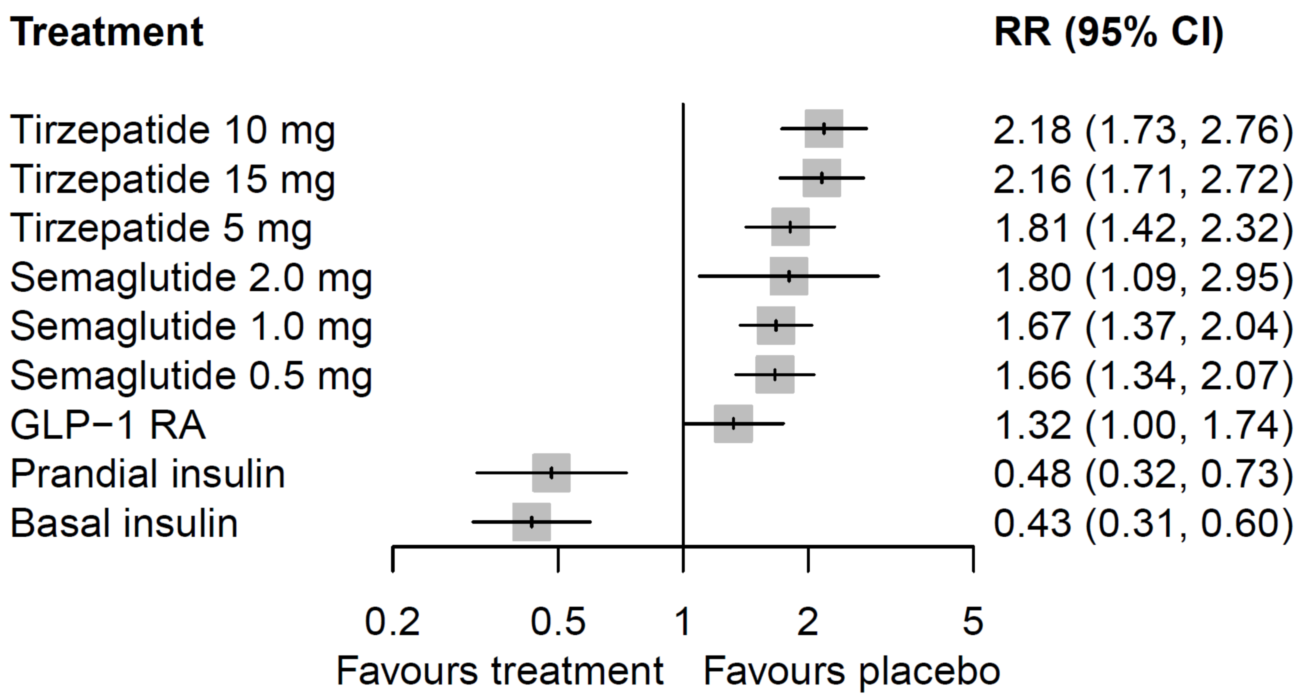

ESM Fig. 12 Network meta-analysis results compared to placebo for the incidence of serious adverse events. RR, risk ratio

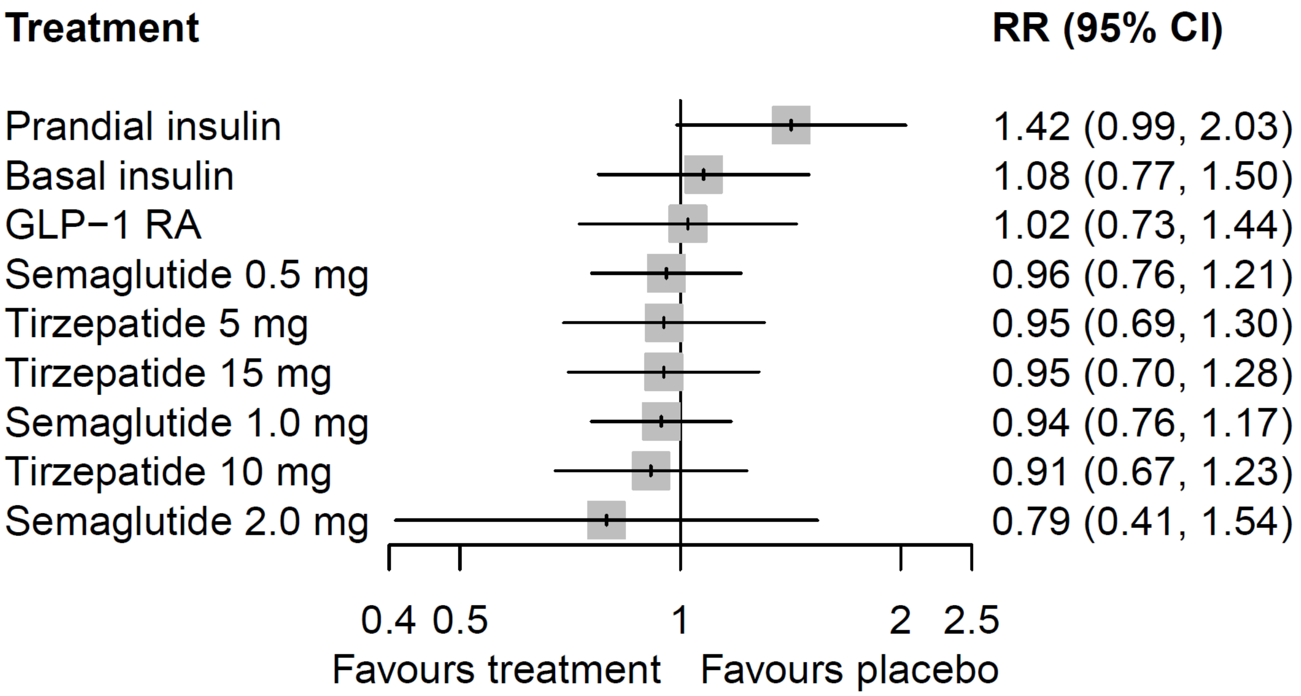

Supplement: Supplementary file 1 — Supplementary file1 (PDF 2326 KB) [file 125_2024_6144_MOESM1_ESM.pdf]
